# Yeast genetic interaction screen of human genes associated with amyotrophic lateral sclerosis: identification of MAP2K5 kinase as a potential drug target

**DOI:** 10.1101/gr.211649.116

**Published:** 2017-09

**Authors:** Myungjin Jo, Ah Young Chung, Nozomu Yachie, Minchul Seo, Hyejin Jeon, Youngpyo Nam, Yeojin Seo, Eunmi Kim, Quan Zhong, Marc Vidal, Hae Chul Park, Frederick P. Roth, Kyoungho Suk

**Affiliations:** 1 Department of Pharmacology, Brain Science and Engineering Institute, and Department of Biomedical Sciences, BK21 Plus KNU Biomedical Convergence Program, Kyungpook National University School of Medicine, Daegu, 41944, Korea;; 2 Department of Biomedical Sciences, Korea University Ansan Hospital, Ansan-si, Gyeonggi-do, 425-707, Korea;; 3 Donnelly Centre and Departments of Molecular Genetics and Computer Science, University of Toronto and Lunenfeld-Tanenbaum Research Institute, Sinai Health System, Toronto, Ontario M5G 1X5, Canada;; 4 Department of Biological Sciences, Wright State University, Dayton, Ohio 45435, USA;; 5 Center for Cancer Systems Biology (CCSB) and Department of Cancer Biology, Dana-Farber Cancer Institute, Boston, Massachusetts 02215, USA;; 6 Canadian Institute for Advanced Research, Toronto, Ontario M5G 1Z8, Canada

## Abstract

To understand disease mechanisms, a large-scale analysis of human–yeast genetic interactions was performed. Of 1305 human disease genes assayed, 20 genes exhibited strong toxicity in yeast. Human–yeast genetic interactions were identified by en masse transformation of the human disease genes into a pool of 4653 homozygous diploid yeast deletion mutants with unique barcode sequences, followed by multiplexed barcode sequencing to identify yeast toxicity modifiers. Subsequent network analyses focusing on amyotrophic lateral sclerosis (ALS)-associated genes, such as optineurin (*OPTN*) and angiogenin (*ANG*), showed that the human orthologs of the yeast toxicity modifiers of these ALS genes are enriched for several biological processes, such as cell death, lipid metabolism, and molecular transport. When yeast genetic interaction partners held in common between human OPTN and ANG were validated in mammalian cells and zebrafish, MAP2K5 kinase emerged as a potential drug target for ALS therapy. The toxicity modifiers identified in this study may deepen our understanding of the pathogenic mechanisms of ALS and other devastating diseases.

Genetic mutations, such as insertions, deletions, and point mutations, are often associated with dysfunctions of cellular differentiation, proliferation, migration, or cell death, thereby increasing susceptibility to common disorders (Childs and Valle 2000; Jimenez-Sanchez et al. 2001). However, for most genetic disorders, it is not known how a particular mutation causes the pathological condition. To address this issue, researchers have used genomic information (Hofker et al. 2014) and network-based approaches (Linghu et al. 2009; Barabási et al. 2011; Vidal et al. 2011). Yeast, as a systems biology model, is a simple and genetically tractable eukaryotic organism (Botstein and Fink 2011). High-throughput methods have been useful in identifying yeast genetic interactions including, for example, synthetic lethal interactions, in which deletion of two nonessential genes together is lethal. This method has generated a large-scale genetic interaction network (Tong et al. 2001, 2004; Costanzo et al. 2010, 2016). Similarly, genetic interactions between yeast and human genes have been used to predict the pathological functions of disease genes. Neurodegenerative diseases are often associated with protein misfolding (Forman et al. 2004; Frost and Diamond 2012; Valastyan and Lindquist 2014). Cytoplasmic inclusions containing tau, transactive response DNA-binding protein (TARDBP), superoxide dismutase (SOD), and synuclein alpha are the pathological hallmarks of neurodegenerative diseases. The cellular mechanism of neurodegenerative diseases such as Parkinson's disease (Outeiro and Lindquist 2003; Cooper et al. 2006; Chung et al. 2013; Tardiff et al. 2013; Caraveo et al. 2014), Huntington's disease (Krobitsch and Lindquist 2000; Meriin et al. 2002; Duennwald et al. 2006a,b), Alzheimer's disease (Bagriantsev and Liebman 2006), Creutzfeldt–Jakob disease (Ma and Lindquist 1999), and amyotrophic lateral sclerosis (ALS) (Johnson et al. 2008) have been studied in the yeast system. Abnormal expression of disease-associated proteins such as TARDBP, huntingtin, and synuclein alpha leads to the formation of toxic cytoplasmic aggregates in yeast cells, and the deletion of particular yeast genes modulates human gene-induced toxicity (Giorgini et al. 2005; Cooper et al. 2006; Gitler 2008; Johnson et al. 2008; Kryndushkin et al. 2012). These features have been used for genome-wide screens of genetic interactions of human disease genes. However, previous studies of human–yeast genetic interactions have been performed using an array format, which is laborious and time-consuming. Efficient methods for genome-wide screens of human–yeast genetic interactions are needed.

ALS, also known as Lou Gehrig's disease, is a neurodegenerative disease involving motoneuron loss in the cerebral cortex, brainstem, and spinal cord (Cleveland and Rothstein 2001; Ferraiuolo et al. 2011). Riluzole is the only drug that has been approved by the US Food and Drug Administration for ALS therapy; several therapeutic agents are in clinical trials (Sreedharan and Brown 2013). Mutations in several genes, including *SOD1*, *TARDBP*, FUS RNA binding protein (*FUS*), valosin containing protein (*VCP*), FIG4 phosphoinositide 5-phosphatase (*FIG4*), optineurin (*OPTN*), angiogenin (*ANG*), and ubiquilin 2 (*UBQLN2*), are associated with familial and sporadic ALS (Greenway et al. 2006; Van Damme and Robberecht 2009; Maruyama et al. 2010; Ticozzi et al. 2011). OPTN is an adaptor protein that interacts with RAB8A (also known as RAB8) (del Toro et al. 2009), huntingtin (Anborgh et al. 2005; del Toro et al. 2009), myosin VI (Sahlender et al. 2005), TANK binding kinase 1 (Gleason et al. 2011), and the transferrin receptor (Nagabhushana et al. 2010). OPTN has been implicated in membrane trafficking (Sahlender et al. 2005; Au et al. 2007; Nagabhushana et al. 2010) and signaling to induce NF-κB activation (Zhu et al. 2007; Nagabhushana et al. 2011). Overexpression of wild-type and mutant OPTN induces the death of ocular cell types (Park et al. 2006; Shen et al. 2011). OPTN is also an autophagy receptor that mediates the clearance of cytosolic *Salmonella* (Wild et al. 2011). *ANG*, another ALS-linked gene, plays roles in tumor angiogenesis (Pavlov and Badet 2001; Kishimoto et al. 2005), processing of ribosomal RNA (Tsuji et al. 2005), and neuroprotection (Kieran et al. 2008; Sebastia et al. 2009). Recent studies have indicated that ANG is a stress-activated RNase. ANG-cleaved tRNA fragments inhibit translation initiation and promote stress granule assembly (Emara et al. 2010; Ivanov et al. 2011; Skorupa et al. 2012). Wild-type ANG reduces the death of motoneurons in an ALS mouse model (Kieran et al. 2008), whereas mutant ANG or knockdown of ANG promotes hypoxia-induced cell death (Kieran et al. 2008; Subramanian et al. 2008; Sebastia et al. 2009). In addition, ANG plays a protective role in a mouse model of Parkinson's disease (Steidinger et al. 2011). Despite recent progress in the field, the molecular and cellular bases for neurodegeneration in ALS are not yet established. Understanding how ALS-linked genes modulate motoneuron degeneration is a key for understanding ALS pathology and protecting motoneurons from damage during disease progression. Here, to better understand disease pathways, we performed a human–yeast genetic interaction screen for human disease genes in a pool format. Subsequent studies focused on ALS-associated genes (*OPTN* and *ANG*) and their toxicity modifiers.

## Results

### Selection of OMIM genes that induce strong toxicity in yeast

**Figure 1.**
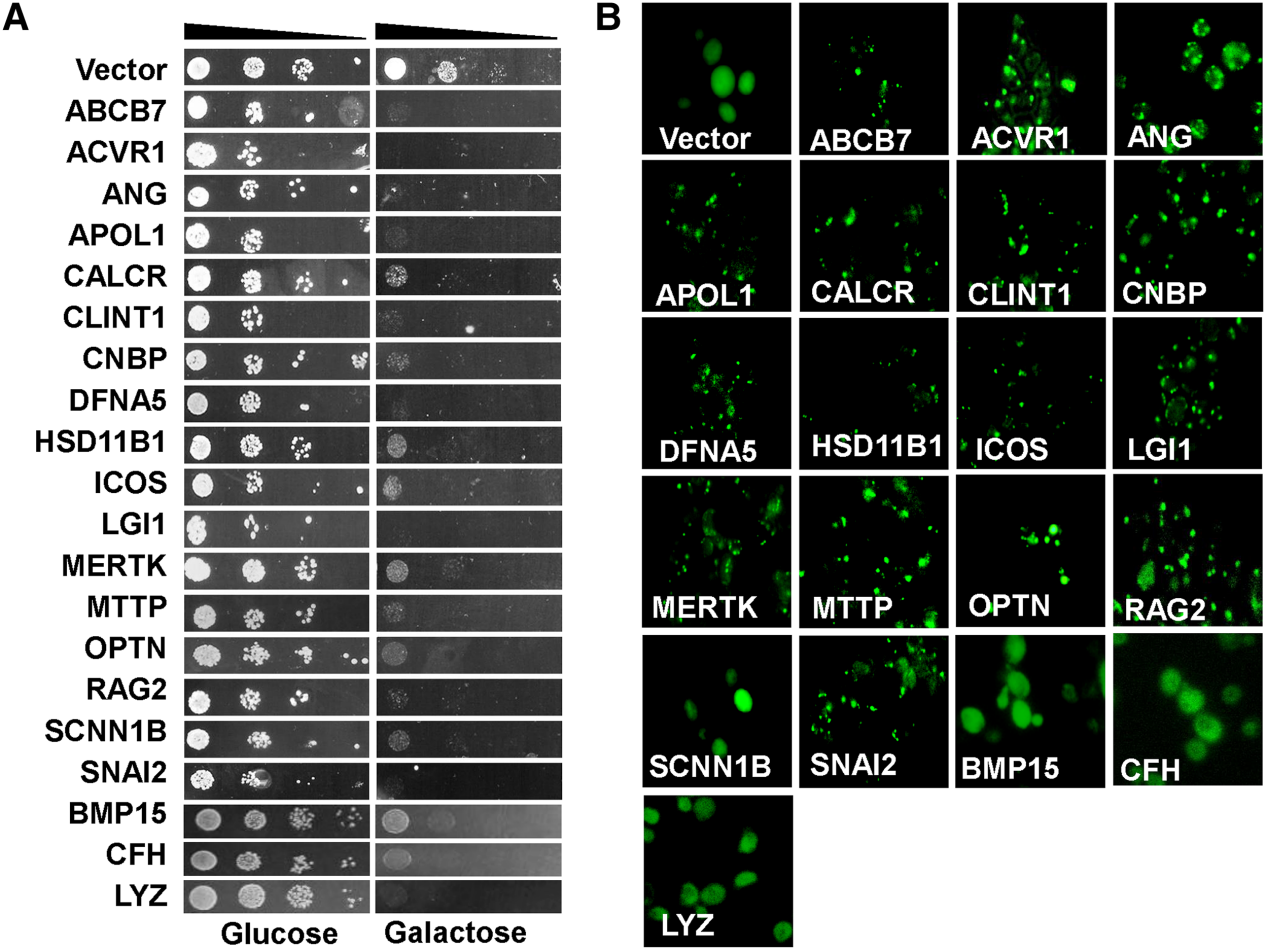
Overexpression of selected OMIM genes induces toxicity and cytoplasmic aggregates in yeast. (*A*) In total, 1305 OMIM ORFs were cloned under the control of a galactose-inducible promoter in pAG425 vectors. pAG425Gal-OMIMs were individually transformed into yeast. Transformants were grown in SRaf-Leu medium for 16 h, spotted onto SD-Leu agar plates (OMIM expression “off”) or SGal-Leu agar plates (OMIM expression “on”), and incubated for 3 d. Shown are 10-fold serial dilutions starting with an equal number of cells expressing the 20 toxic OMIM genes. Nontoxic OMIM genes are not shown. (*B*) Yeast cells expressing the “C-terminal GFP-tagged OMIM” fusion proteins were visualized with fluorescence microscopy. GFP alone was distributed in the cytoplasm. Most of the toxic GFP-tagged OMIM proteins formed protein aggregates.

OMIM is a compendium of human genes and genetic phenotypes for all known Mendelian disorders. OMIM focuses on the relationship between phenotype and genotype. However, disease mechanisms are not clearly understood for most human disease genes. Gene or protein interaction networks for the disease genes may improve understanding of disease mechanisms (Barabási et al. 2011; Vidal et al. 2011). The *S. cerevisiae* genome has been sequenced in its entirety (Goffeau et al. 1996), and genetic interactions have been profiled for nearly all genes (Costanzo et al. 2016). This knowledge base can be used for functional analysis. In our high-throughput, toxicity-based screens of genetic interactions between human disease genes and yeast genes, the toxicity of human OMIM genes was first evaluated in yeast. OMIM ORFs were cloned into pAG425Gal-ccdB under the control of the *GAL1* promoter to enable inducible overexpression in yeast. We selected a set of 1305 genes associated with human disorders in OMIM from human ORFeome collections—ORFeome 1.1 (Rual et al. 2004), ORFeome 3.1 (Lamesch et al. 2007), and ORFeome 7.1 (http://horfdb.dfci.harvard.edu/hv7). Spotting assays on SGal-Leu agar plates were performed to determine the toxicity of OMIM ORFs in yeast. Of 1305 human OMIM genes tested, 20 OMIM ORFs induced strong toxicity when expressed in yeast (Fig. 1A). Among the 20 OMIM ORFs, yeast toxicity of OPTN was consistent with a previous report (Kryndushkin et al. 2012). Unfortunately, several human disease genes known to be toxic in yeast, e.g., *TARDBP*, *FUS*, and *HNRNPA1*, were not included in the initial human ORFeome collections that we used for screening. The 20 OMIM ORFs that were highly toxic in yeast were subjected to further study; OMIM ORFs with modest toxicity were excluded. The DNA sequences of expression clones for the 20 OMIM ORFs were verified by Sanger sequencing. Because several human proteins associated with neurodegenerative diseases, such as synuclein alpha and TARDBP, were found to form cytoplasmic aggregates in yeast in previous studies (Gitler 2008; Johnson et al. 2008), we hypothesized that the toxicity of the 20 human genes was linked to the formation of protein aggregates in yeast. To test this hypothesis, the 20 human genes were expressed in yeast as GFP-fused proteins, which were then visualized under a fluorescence microscope. Of the 20 human genes, 16 genes induced the formation of protein aggregates when expressed in yeast. Protein aggregation was not observed for four genes: sodium channel epithelial 1 beta subunit; bone morphogenetic protein 1; complement factor H; and lysozyme (Fig. 1B). Interestingly, most of the OMIM genes tested, regardless of their previous association with neurodegenerative disease, formed toxic aggregates. These characteristics can be used for high-throughput genetic or chemical screens of toxicity modifiers. The 20 OMIM genes that induced toxicity in yeast are listed with their associated disease phenotypes in Table 1. The formation of GFP-fused protein foci, however, might not necessarily indicate aggregation and might indicate subcellular localization, such as organelle, vesicle, or nonmembrane bound compartment. Nevertheless, a strong toxicity of the human genes in yeast precluded further characterization of the aggregation via a biochemical technique.

**Table 1.**
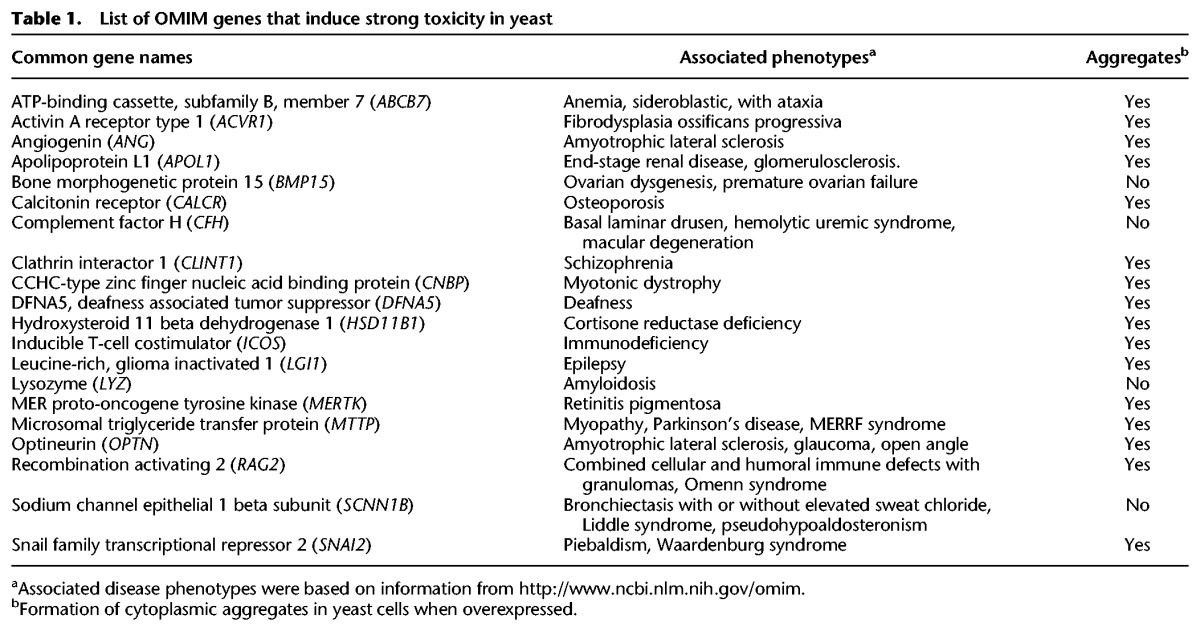
List of OMIM genes that induce strong toxicity in yeast

### Identification of human–yeast genetic interactions using a genome-wide pooled screen in yeast

**Figure 2.**
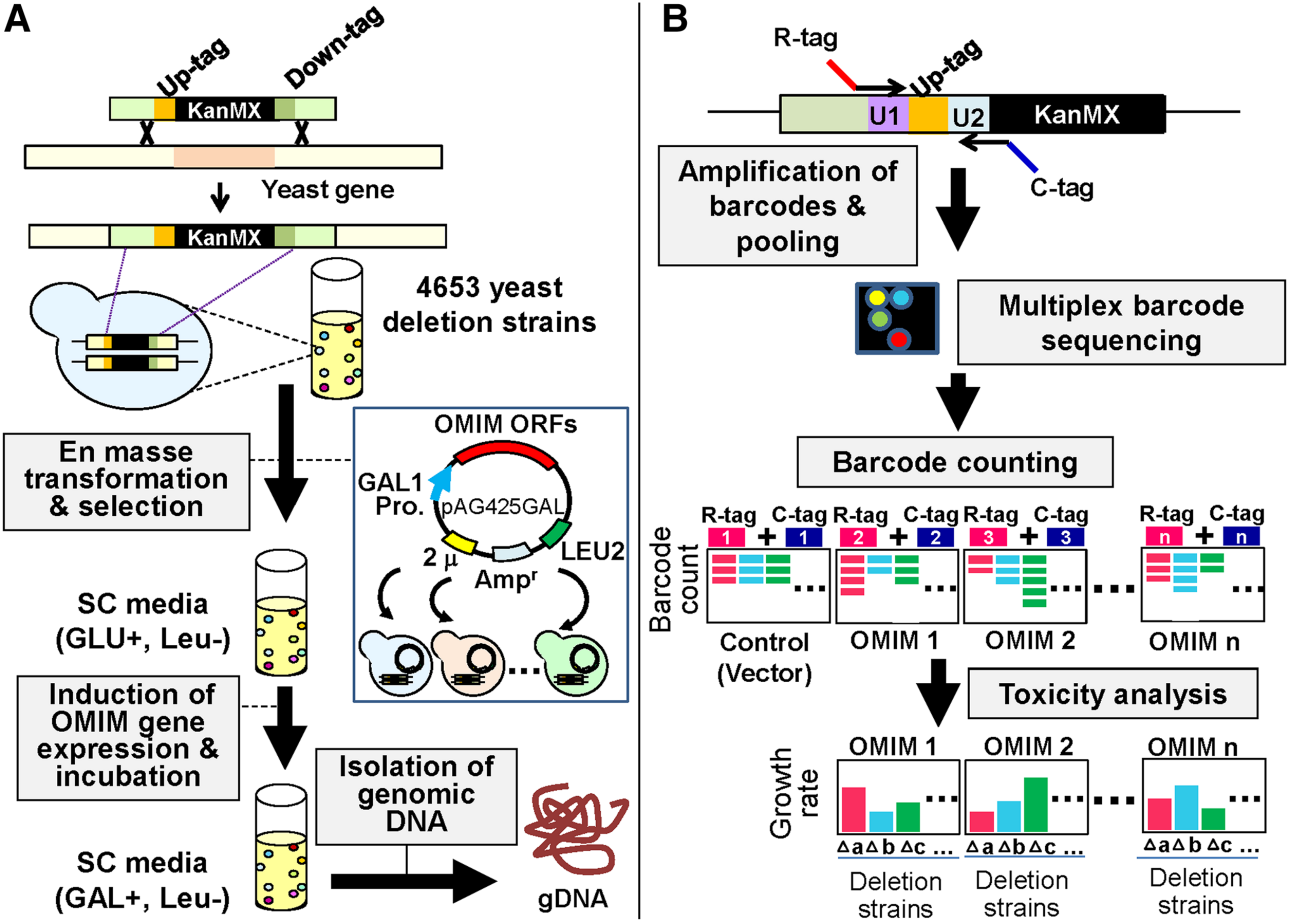
Flowchart describing the yeast genetic interaction screen. (*A*) The 20 toxic OMIM genes were transformed individually into a pool of 4653 yeast homozygous deletion strains containing a 20-bp DNA barcode sequence. Transformants were selected in SD-Leu medium for 16 h and then washed twice with PBS. The cells were resuspended in SGal-Leu medium and incubated for 2 d to induce the expression of OMIM genes under the control of the *GAL1* promoter. Genomic DNA was separately isolated from cells harvested at the end of pooled culture in the presence of GLU or GAL. (*B*) Barcodes were amplified from genomic DNA with multiplexed primers containing distinct combinations of two different tags for each OMIM gene. Equal amounts of DNA amplified for each OMIM gene were pooled and subjected to multiplex barcode sequencing using an Illumina Genome Analyzer. Next-generation sequencing data were then analyzed for barcode counting.

To identify human–yeast genetic interactions for the 20 toxic OMIM genes, we performed a genome-wide pooled screen, in which genetic interactions were identified from toxicity modification in a pooled and multiplexed format (Fig. 2). In this screen, individual OMIM genes were first introduced into yeast deletion pools with unique barcode sequences. Second, OMIM gene expression was turned on in yeast. Each yeast deletion pool containing a single OMIM gene was cultured individually. Third, yeast barcodes were separately amplified from the deletion pool culture for each individual OMIM gene. Finally, yeast barcode abundance was quantified using multiplexed next-generation sequencing to identify OMIM-yeast genetic interactions. Change in yeast barcode abundance indicates the differential growth of a deletion strain, which reflects the modulation of OMIM gene toxicity in the absence of a specific yeast gene (Smith et al. 2009, 2010).

**Figure 3.**
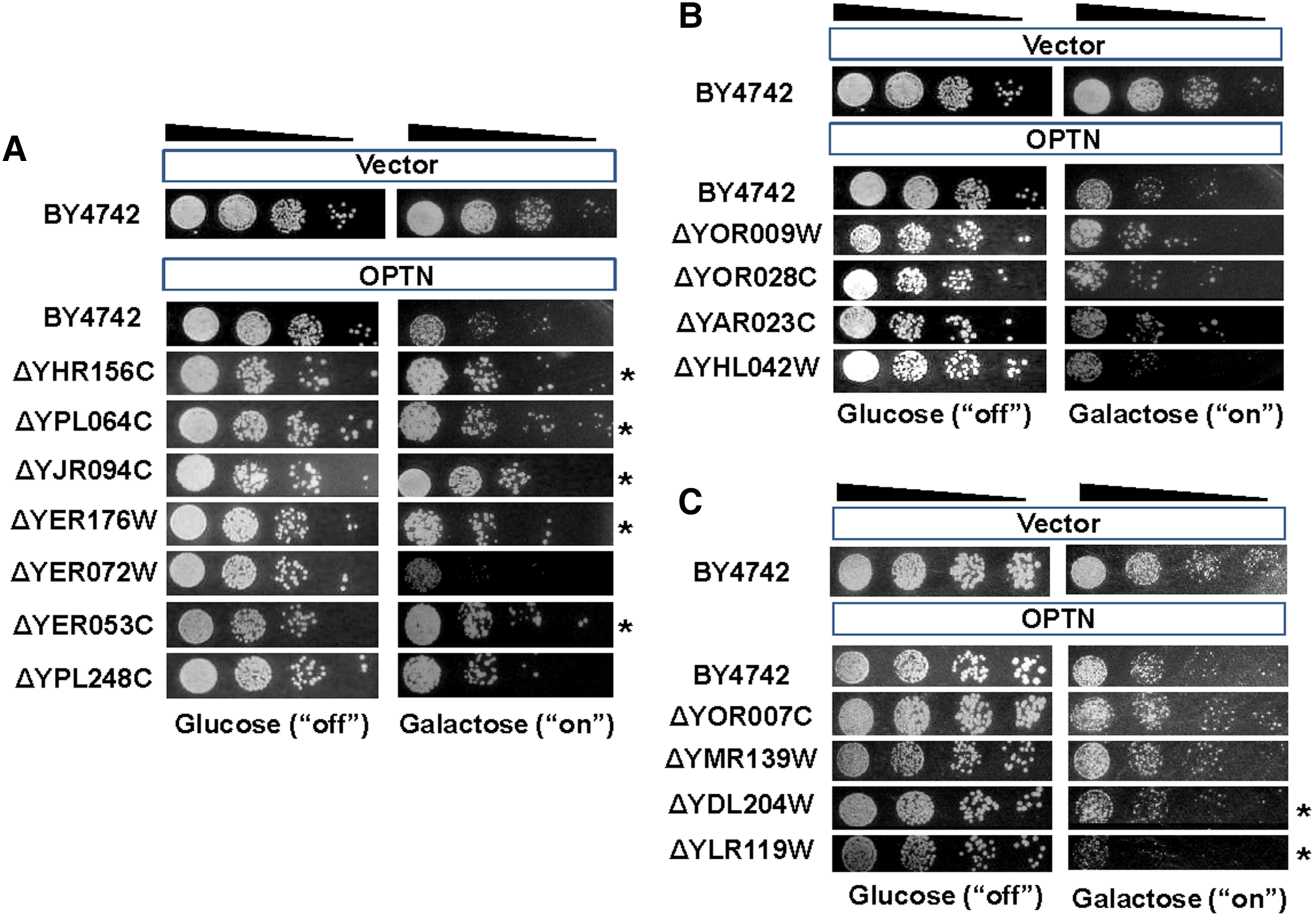
Validation of the genome-wide screening data for OPTN. Barcode counting was used to screen OMIM-yeast genetic interactions. From the Bar-seq data analysis and *Z*-score distributions, three groups of yeast genes were chosen for spotting assays: group 1, toxicity suppressors; group 2, no effects; and group 3, toxicity enhancers. OPTN toxicity was analyzed with spotting assays in the wild-type yeast strain (BY4742), toxicity-suppressing gene deletion strains (*A*), no-effect gene deletion strains (*B*), and toxicity-enhancing gene deletion strains (*C*). pAG425GAL-ccdB was used as the empty vector control. Twenty-four yeast deletions were tested. Representative results are shown. Asterisks indicate yeast deletions that suppressed or enhanced OPTN toxicity.

Using this method, we obtained a list of OMIM-yeast genetic interactions for the 20 OMIM genes (Supplemental Table S1). The toxicity modifier list was obtained using the normalized Bar-seq data. Toxicity suppressors and enhancers were identified from the corrected *Z*-score, and the toxicity modifiers were divided into three groups: toxicity suppressors, *Z*-score >1.96; toxicity enhancers, *Z*-score <−1.96; and no-effect, yeast deletions that were not suppressors or enhancers (Supplemental Table S2). Suppression or enhancement of OMIM toxicity by deletion of a specific yeast gene was tested using individual yeast deletion strains in order to validate the results of the large-scale OMIM-yeast genetic interaction screen. Several toxicity modifiers were selected from each group in the OPTN modifier list. Modification of OPTN toxicity was tested in the selected yeast deletion strains. When OPTN was individually expressed in the selected deletion strains, the results of the spot assay and the genome-wide screen were consistent for 75% of the genes in the suppressor group and 62.5% of those in the no-effect group. However, consistency between the pooled assay and spot assay was low for the enhancer list (12.5%). Representative images of the OPTN spot assay are shown in Figure 3. The galactose-induced toxicity of OPTN was modified in specific yeast deletion strains. The toxicity and protein aggregation patterns of GFP-tagged OPTN proteins and untagged proteins were similar when assessed in several yeast deletion strains (Supplemental Fig. S1). A similar validation experiment was conducted for the small number of CLINT1 modifiers (consistency for the suppressor list = 62.5%; no-effect group = 62.5%; enhancer list = 25%). The validation experiment was further extended to 10% of randomly selected modifying genes from toxicity suppressors and enhancers for the three OMIM genes (*OPTN*, *ANG*, and *CLINT1*) (Supplemental Table S3; Supplemental Fig. S2). In the large-scale validation experiments, average consistency for the three OMIM genes was increased from 68.7% to 77.9% for toxicity suppressors. High inconsistencies were still observed for toxicity enhancers (average consistency 24.4%). Thus, the validity of the genome-wide genetic interaction screen using toxicity modification and Bar-seq appeared to be limited to toxicity suppressors, possibly because query gene–induced toxicity was too strong to detect toxicity enhancers. Therefore, OMIM-yeast genetic interactions were defined by the detection of yeast gene deletions suppressing the yeast toxicity of a given human OMIM ORF.

**Figure 4.**
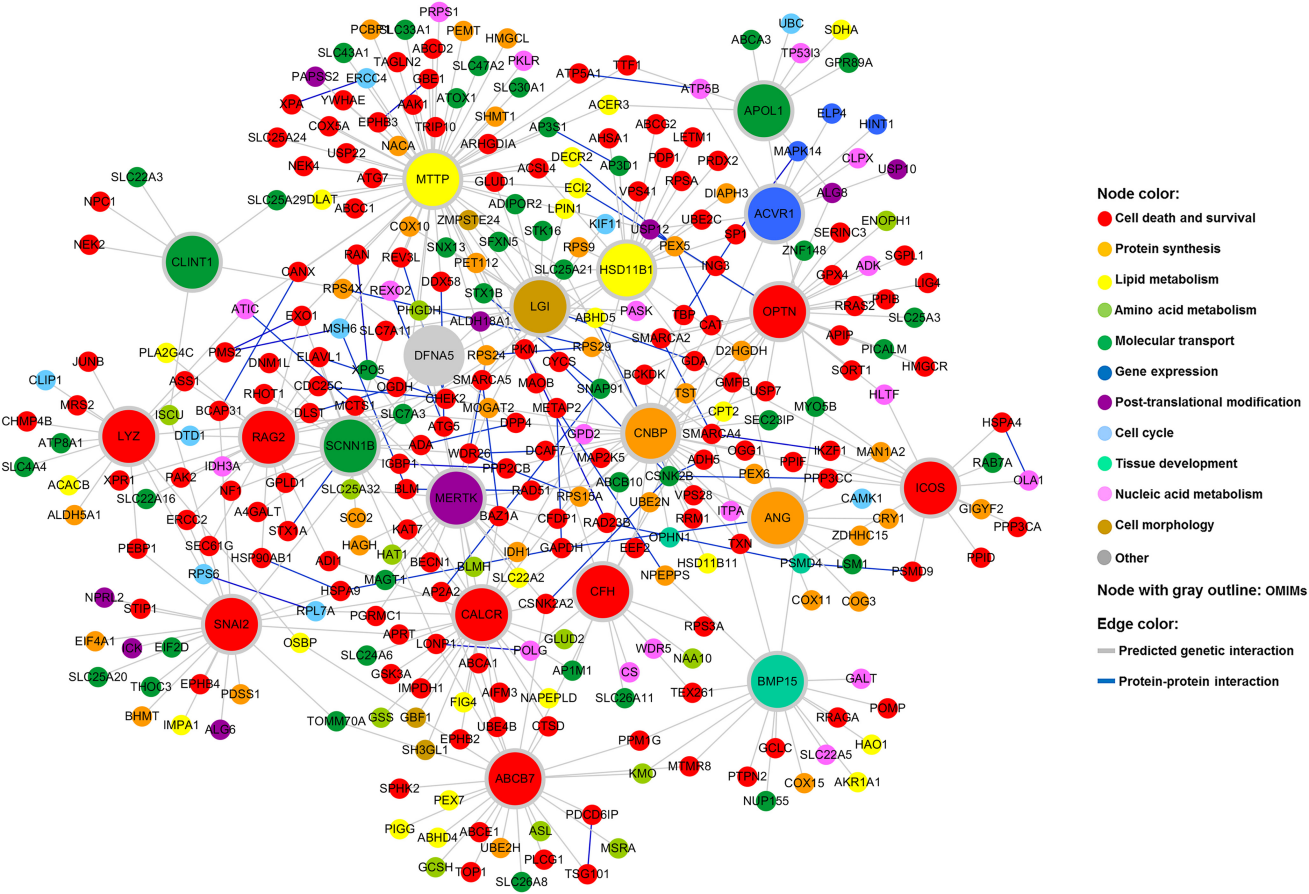
Human–yeast genetic interaction network. Human orthologs of yeast genes whose deletion suppressed the toxicity of the 20 OMIM ORFs were identified. A network view of these human orthologs was generated using Cytoscape. The node color corresponds to the biological function category to which the gene belongs. The color of an edge indicates the type of interaction.

To identify OMIM–human gene interactions, human orthologs of the yeast toxicity suppressors were found using the Karolinska Institute's InParanoid Database (http://inparanoid.sbc.su.se) and NCBI's HomoloGene (http://www.ncbi.nlm.nih.gov/homologene) (Supplemental Table S4). An OMIM–human gene interaction network was constructed using the human orthologs of yeast toxicity suppressors for the 20 OMIM genes (Fig. 4). This network also revealed the relationships among the 20 OMIM genes. IPA-based protein–protein interactions were also included in the network. Thus, a genome-wide human–yeast genetic interaction screen followed by a search for human orthologs provided a “first draft” disease-gene interaction network for a subset of human disease genes.

### Genetic interactions between ALS-associated genes and yeast toxicity modifiers

**Figure 5.**
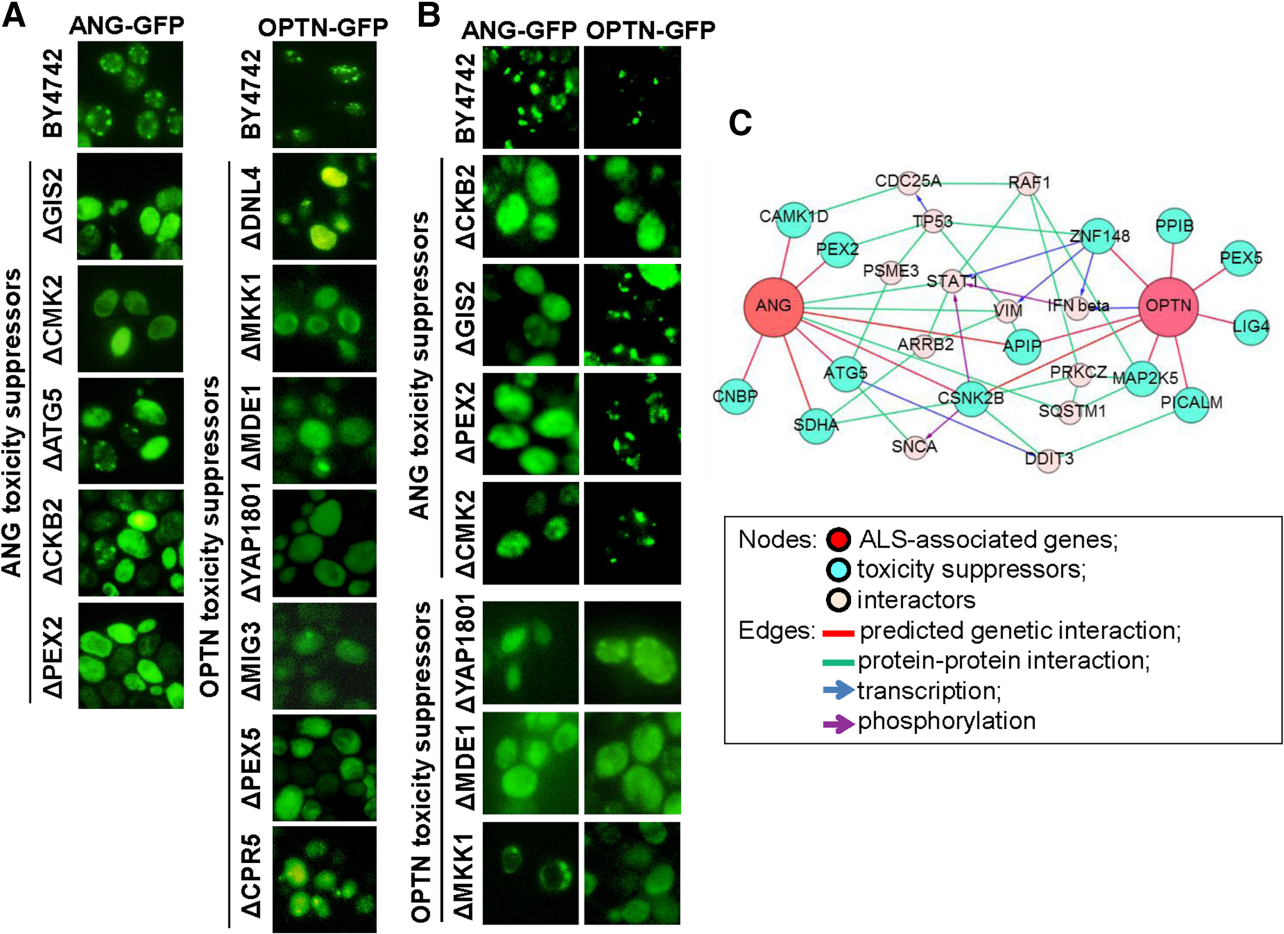
Formation of ALS-associated protein aggregates is attenuated in specific yeast gene deletion strains. (*A*) Loss of specific yeast genes reversed the aggregation of the ALS-associated proteins. To observe protein localization, OPTN and ANG were cloned into pAG425GAL-ccdB-GFP and transformed into the deletion strains. Cells expressing OPTN-GFP or ANG-GFP were observed with fluorescence microscopy after 16 h of *OPTN*/*ANG* gene induction. (*B*) OPTN and ANG protein aggregation was cross-tested in representative yeast deletion strains of ANG and OPTN toxicity suppressors, respectively. (*C*) From the genetic interaction results of the cross-test, a network for the two ALS genes was constructed.

Among the 20 OMIM genes whose genetic interactions were analyzed in yeast, *OPTN* and *ANG* have been commonly linked to familial ALS (Van Damme and Robberecht 2009; Ticozzi et al. 2011), and subsequent studies focused on these two ALS-associated genes. The other 18 genes were associated with all different diseases without common phenotype. This was our rationale for focusing on ALS. Other ALS genes like *TARDBP*, *SOD1*, *VCP*, and *C9orf72* were not included in our human ORFeome collections used for the initial screen. To analyze the genetic interactions of these two OMIM genes in a mammalian system, human orthologs of yeast genes whose deletion suppressed OPTN- and ANG-induced toxicity were functionally categorized. IPA analysis revealed that the human orthologs were enriched for several biological functions, such as cell death/survival, lipid metabolism, molecular transport, engulfment of cells, and protein kinase cascade (Table 2). Interestingly, the list of human orthologs included other OMIM genes, indicating a functional connection between the ALS-associated genes and other OMIM genes. This was also evident in the subnetwork that focused on *OPTN* and *ANG* (Supplemental Fig. S3). Next, we used individual yeast deletion strains to test the genetic interactions between the ALS-associated OMIM genes (*OPTN* and *ANG*) and yeast genes whose human orthologs were enriched for different biological functions (Table 2). The formation of protein aggregates was examined in yeast deletion strains overexpressing OPTN or ANG. Deletion of specific yeast genes attenuated the formation of OPTN or ANG protein aggregates (Fig. 5A). The yeast deletions, their human orthologs, and the functional categories are listed in Table 3. We found that deletion of four yeast genes (*CKB2*, *YAP1801*, *MDE1*, and *MKK1*) attenuated the formation of both OPTN and ANG protein aggregates (Fig. 5B), indicating cross-interaction between OPTN/ANG and the four yeast genes. MKK1 deletion completely reversed the OPTN protein aggregation. Although the effect of MKK1 deletion on ANG was less pronounced, it appeared to partly attenuate the ANG protein aggregation as well. Genetic interaction of ANG was further validated using yeast spotting assay (Supplemental Fig. S4). From these results, we constructed a predicted network of OPTN/ANG-human genetic interactions (Fig. 5C). The predicted network also included protein–protein interactions, transcriptional regulation, and phosphorylation. The two ALS-associated genes, *OPTN* and *ANG*, shared many genetic and protein interaction partners.

**Table 2.**
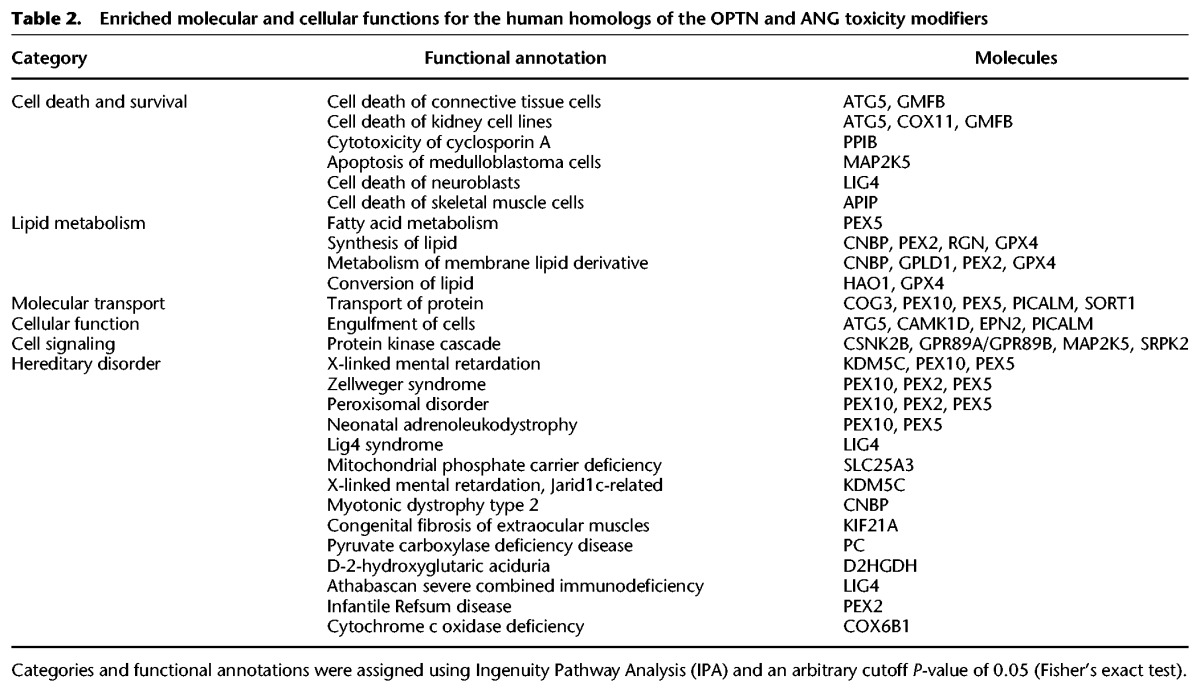
Enriched molecular and cellular functions for the human homologs of the OPTN and ANG toxicity modifiers

**Table 3.**
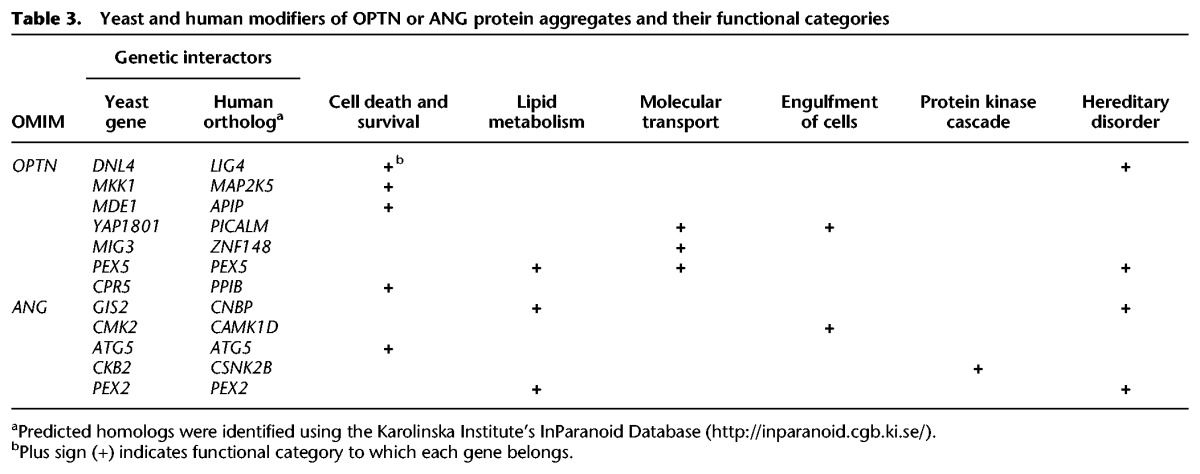
Yeast and human modifiers of OPTN or ANG protein aggregates and their functional categories

### Genetic interaction of ALS-associated genes in mammalian cells

**Figure 6.**
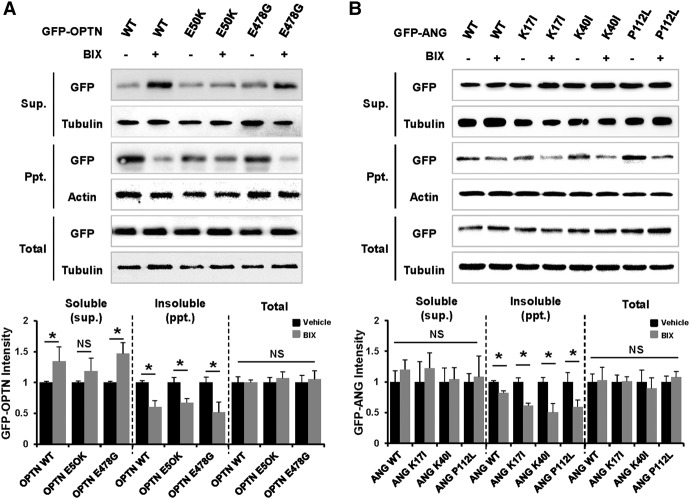
MAP2K5 inhibition attenuates the formation of OPTN and ANG protein aggregates in mammalian cells. NIH3T3 cells were transiently transfected with wild-type GFP-OPTN or GFP-OPTN mutants (E50K or E478G) (*A*) and wild-type GFP-ANG or GFP-ANG mutants (K17I, K40I, or P112L) (*B*). At 36 h after transfection, cells were incubated with vehicle or 10 µM BIX 02189 (MAP2K5 inhibitor) for 12 h. Cells were lysed in NP40 lysis buffer, and the lysates were separated into soluble and insoluble fractions: (Sup.) supernatant; (Ppt.) precipitate. OPTN or ANG proteins in the cellular fractions were detected with Western blot analysis using an antibody against GFP. DMSO (0.1% v/v) was used as a vehicle. The densitometry analysis was plotted as an intensity ratio of soluble GFP-OPTN/tubulin (Sup.), insoluble GFP-OPTN/actin (Ppt.), and total GFP-OPTN/tubulin (Total). The densitometry analysis for GFP-ANG was done in the same manner. The results of the densitometric analysis (*bottom*) are presented as the mean ± SD (*n* = 3); (*) *P* < 0.05 versus vehicle.

Genetic interactions between the ALS-associated genes and the human orthologs of the yeast toxicity modifiers were investigated in mammalian cells in culture. The studies in mammalian cells focused on MAP2K5 (human ortholog of yeast MKK1, a common genetic interaction partner for OPTN and ANG), for which a pharmacological inhibitor is commercially available. Because kinases are one of the most exploited therapeutic targets in the current pharmacological research, we focused on MAP2K5 kinase in the subsequent study. Formation of protein aggregates was first examined after transfection with either wild-type or disease-linked variants of OPTN and ANG (Fig. 6). Two OPTN mutants and three ANG mutants were used: OPTN E50K, found in glaucoma patients (Rezaie et al. 2002); OPTN E478G (Maruyama et al. 2010); ANG K17I; ANG K40I; and ANG P112L, found in ALS patients. Inhibition of MAP2K5 using the specific pharmacological inhibitor BIX 02189 reduced the formation of OPTN and ANG aggregates in NIH3T3 cells. The MAP2K5 inhibitor similarly reduced protein aggregates induced by the mutant forms of OPTN and ANG. Similar results were found in fluorescence microscopy analysis of GFP-fused OPTN and ANG proteins (Supplemental Fig. S5). Inhibition of MAPK7 phosphorylation by the MAP2K5 inhibitor BIX 02189 was confirmed with Western blot analysis (Supplemental Fig. S6). We next tested the impact of the candidate modifiers on the expression of the toxic genes. MAP2K5 inhibition did not significantly affect the expression levels of OPTN or ANG (Supplemental Fig. S7), indicating that the deletions that suppressed OPTN or ANG toxicity did not down-regulate the expression of these genes. Given the slight differences in the behavior of WT and disease-causing mutants in mammalian cells, we asked whether there are differences in the toxicity-based genetic interactions between WT and mutants in yeast. *MKK1* deletion that genetically interacted with the WT *OPTN* and *ANG* genes did not interact with disease-causing mutants in the same way (Supplemental Fig. S8). The mutants of OPTN and ANG showed less toxicity in yeast compared with WT, and their genetic interaction with *MKK1* deletion was not as strong as that of WT.

### Genetic interactions of ALS-associated genes in zebrafish

**Figure 7.**
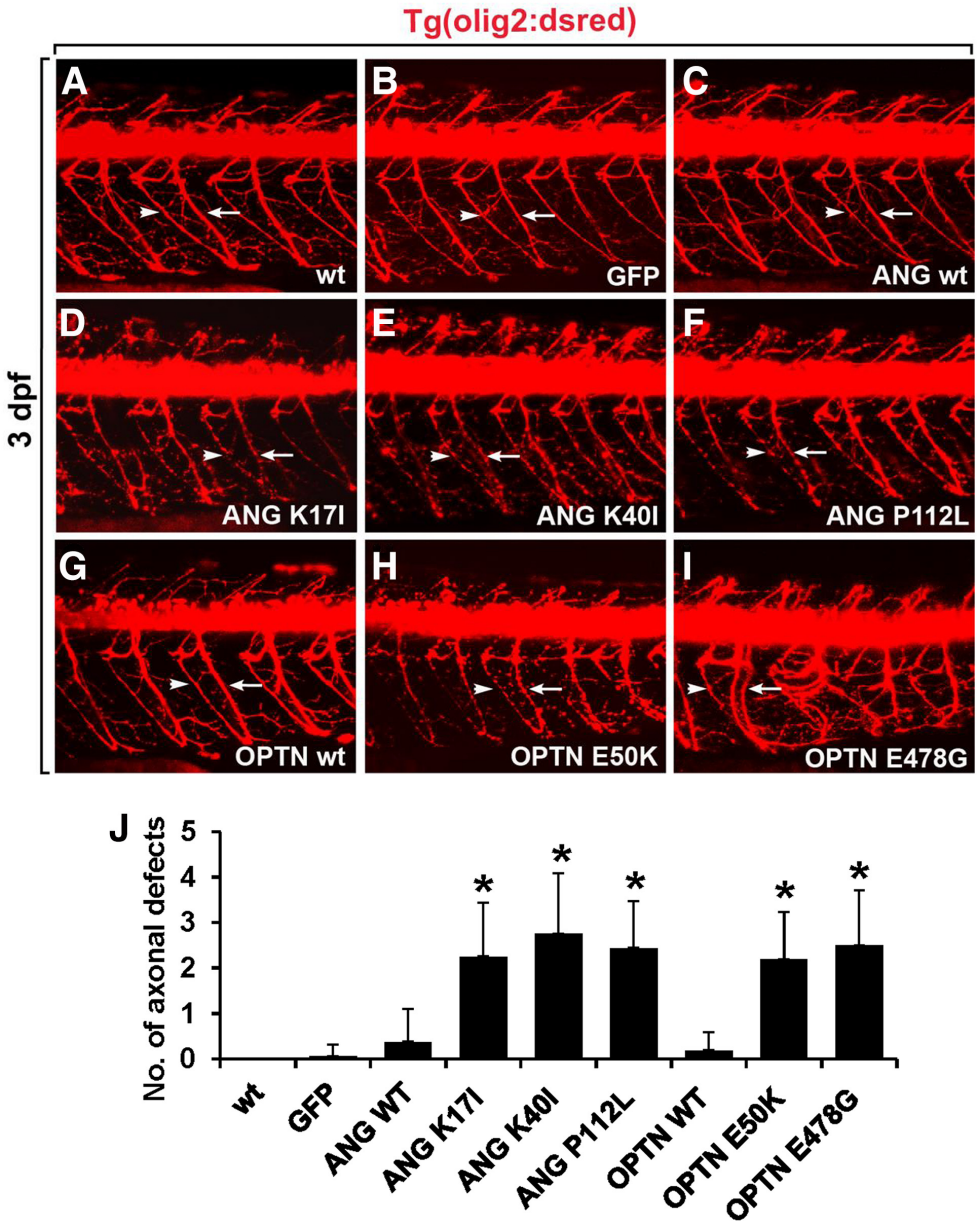
Overexpression of ANG or OPTN mutants causes motor axonopathy in the spinal cord of zebrafish embryo. All panels show lateral views of the spinal cord of *Tg(olig2:dsred2)* embryos, with anterior to the *left* and dorsal to the *top*. Motor axons (arrows) and neuromuscular junctions (NMJs, arrowheads) were detected with DsRed fluorescent protein expression. (*A*,*B*) Visualization of motor axons and neuromuscular junctions in the noninjected (*A*) and *egfp* mRNA-injected control embryos (*B*). (*C*–*F*) Injection of mRNA for wild-type ANG (*C*), ANG K17I (*D*), ANG K40I (*E*), and ANG P112L (*F*) mutants in the *Tg(olig2:dsred2)* embryos. (*G*–*I*) Injection of mRNA for wild-type OPTN (*G*), OPTN E50K (*H*), and OPTN E478G (*I*) mutants in *Tg(olig2:dsred2)* embryos. (*J*) Statistical analysis of *A*–*I*. Axonal defects included axonal swelling and degeneration. Data were obtained from 10 control and 10 mRNA-injected embryos. (*) *P* < 0.05 versus GFP-expressing control embryos; mean ± SD.

We used a zebrafish model to evaluate the genetic interaction between the ALS-associated genes and MAP2K5. Zebrafish is considered as an excellent vertebrate model for the molecular and genetic dissection of motor neuron disease mechanisms (Babin et al. 2014). In many neurodegenerative diseases, both “gain of function” and “loss of function” of disease genes are thought to be important for pathogenesis. Disease-causing mutations often lead to problems with protein homeostasis (proteostasis) and ensuing protein aggregate formation. Thus, overexpression of wild-type or mutant forms of disease genes may mimic the proteostasis aspect of diseases. Ectopic expression of the disease-linked mutant forms of OPTN or ANG resulted in motor axonopathy in the zebrafish embryos. This was tested by injecting *OPTN* or *ANG* mRNAs into *Tg(olig2:dsred2)* zebrafish, in which expression of the DsRed fluorescent protein under the control of the *olig2* promoter enabled the detection of spinal motor axons and neuromuscular junctions (NMJs) (Fig. 7; Kucenas et al. 2008). The spinal cords of *Tg(olig2:dsred2)* embryos injected with 300 pg of *ANG* (Fig. 7C) or *OPTN* mRNA (Fig. 7G) showed no significant motor axon phenotype when compared to those of noninjected (Fig. 7A) or *GFP* mRNA-injected (Fig. 7B) control embryos, indicating that overexpression of wild-type ANG or OPTN did not cause motor axonopathy. However, we observed axonopathy including axonal swelling and subsequent degeneration of axons, which are the characteristic phenotypes of motor axons in ALS (Laird et al. 2008; King et al. 2011; Kobayakawa et al. 2015), in the spinal cords of *Tg(olig2:dsred2)* zebrafish upon injection of mRNA for the mutant *OPTN* and *ANG* variants. Motor axon shows axonal swelling and degeneration phenotype in the spinal cord when *Tg(olig2:dsred2)* zebrafish were injected with mRNA for the ANG K17I, ANG K40I, ANG P112L, and OPTN E50K mutants (Fig. 7D–F,H,J), whereas injection of mRNAs for the OPTN E478G mutant caused a significant increase in axon branching and axon disorganization (Fig. 7I,J). We observed similar axonopathy in the spinal cord of *Tg(olig2:dsred2)* zebrafish when transgenic embryos were injected with mRNA for the SOD1 G93A and TARDBP Q331K mutants, which are well-known ALS mutations (Supplemental Fig. S9). The results are consistent with our previous study using the TARDBP Q331K mutant model of ALS (Paik et al. 2015). Altogether, these data indicate that overexpression of mutant variants of ANG and OPTN causes motor axonopathy.

**Figure 8.**
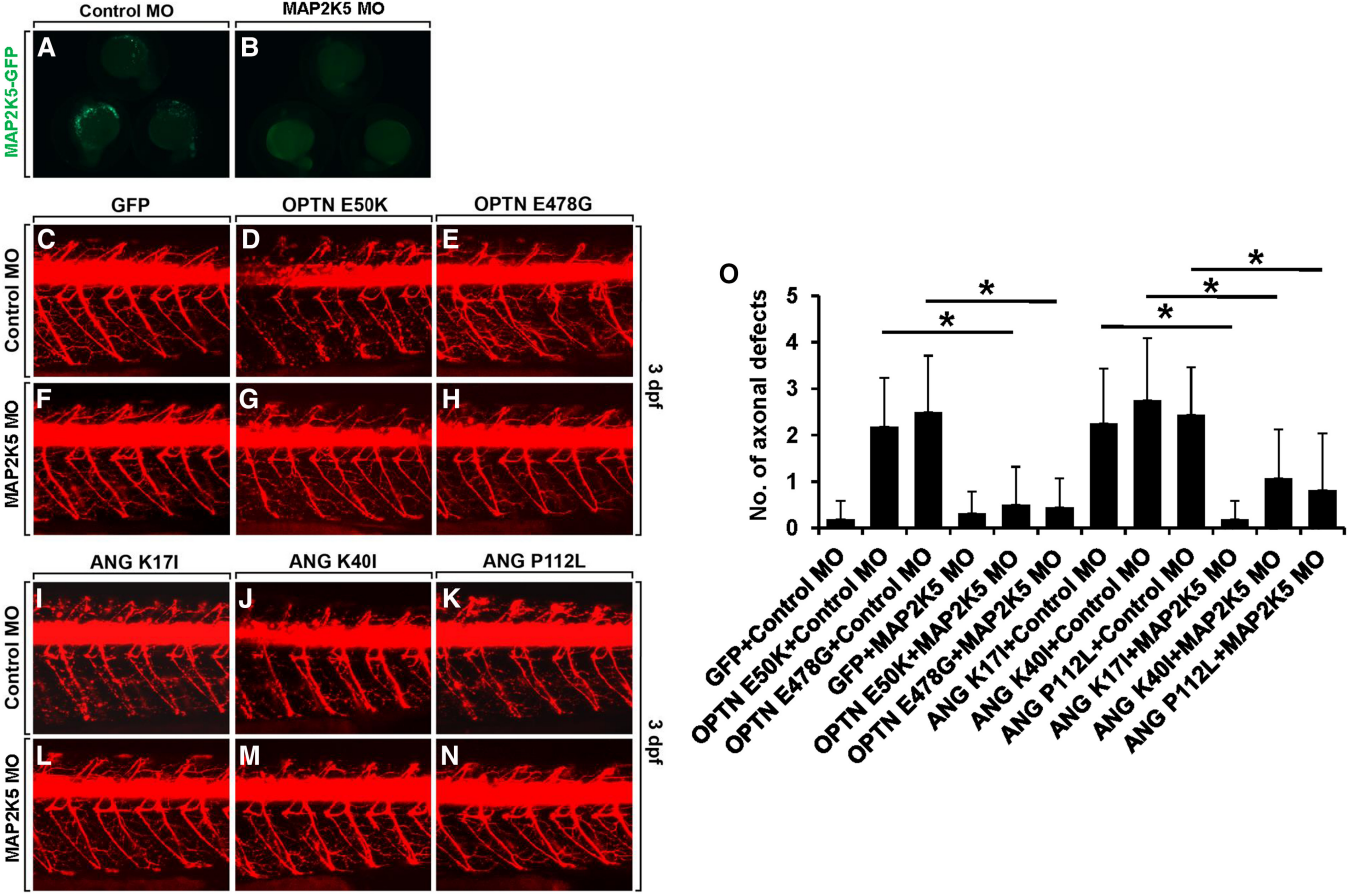
Disease modifying function of MAP2K5 in the zebrafish model of ALS. Knockdown of MAP2K5 rescued mutant OPTN- and ANG-induced motor axonopathy. (*A*,*B*) Expression of EGFP-tagged MAP2K5 protein in embryos injected with scrambled MO (*A*) and *map2k5* MO (*B*) along with the *cmv*:*map2k5*-*egfp* construct. (*C*–*N*) Lateral views of the spinal cords of *Tg(olig2:dsred2)* embryos, with anterior to the *left* and dorsal to the *top*. Motor axons and NMJs were detected from DsRed fluorescent protein expression. (*C*–*H*) The spinal cord of *Tg(olig2:dsred2)* embryos was injected with scrambled MO and GFP (*C*), OPTN E50K (*D*) or OPTN E478G (*E*) mRNA, or injected with *map2k5* MO and GFP (*F*), OPTN E50K (*G*), or OPTN E478G (*H*) mRNA. (*I*–*N*) The spinal cord of *Tg(olig2:dsred2)* embryos was injected with scrambled MO and ANG K17I (*I*), ANG K40I (*J*), or ANG P112L (*K*) mRNA, or injected with *map2k5* MO and ANG K17I (*L*), ANG K40I (*M*), or ANG P112L (*N*) mRNA. (*O*) Statistical analysis of *C*–*N*. Data were obtained from 10 control and 10 mRNA-injected embryos. (*) *P* < 0.05 between the indicated groups; mean ± SD.

The disease modifying function of MAP2K5 was next tested using an antisense morpholino oligonucleotide against *map2k5* (*map2k5* MO), which inhibits *map2k5* expression by blocking its translation (Fig. 8). The specificity of the *map2k5* MO was confirmed by injecting it into zebrafish embryos with a *cmv*:*map2k5*-*efgp* construct, which expressed EGFP-tagged MAP2K5 under the control of the *cmv* promoter. The embryos injected with *map2k5* MO and the *cmv*:*map2k5*-*egfp* construct showed a very low level of EGFP expression (Fig. 8B), whereas the embryos injected with a scrambled MO and *cmv*:*map2k5*-*egfp* exhibited strong EGFP expression (Fig. 8A), demonstrating that the *map2k5* MO inhibited *map2k5*-*egfp* expression. To further quantify the level of morpholino-induced *MAP2K5* knockdown, we used another ectopic MAP2K5 expression system, because there is no antibody available to detect endogenous MAP2K5 protein in zebrafish (Supplemental Fig. S10). We first generated *hsp70*:*map2k5-mCherry* DNA construct, which expresses MAP2K5-mCherry fusion protein under the control of heat-shock inducible promoter (*hsp70*). Next, *hsp70*:*map2k5-mCherry* DNA was injected into the one-cell-stage zebrafish embryos together with either control MO or *MAP2K5* MO. Injected embryos were then heat shocked to induce the expression of exogenous MAP2K5. After heat-shock induction, fluorescence intensity was measured to determine the level of MAP2K5-mCherry fusion protein. *MAP2K5* MO significantly reduced mCherry fluorescence intensity, indicating the effective reduction of MAP2K5 protein expression (Supplemental Fig. S10).

We next investigated the possible disease modifying function of MAP2K5 by injecting *map2k5* MO into zebrafish models of ALS, which were induced by overexpression of OPTN and ANG mutants. The spinal cord of *Tg(olig2:dsred2)* embryos injected with *map2k5* MO and *GFP* mRNA showed no significant motor axonopathy (Fig. 8F), similar to control embryos injected with scrambled MO and *GFP* mRNA (Fig. 8C), indicating that knockdown of *MAP2K5* alone does not cause any phenotypic change. Interestingly, knockdown of *MAP2K5* by *map2k5* MO injection into *Tg(olig2:dsred2)* embryos rescued motor axonopathy induced by OPTN E50K and OPTN E478G overexpression (Fig. 8G,H,O), whereas injection of control MO did not significantly affect the motor axonopathy caused by the overexpression of these mutants (Fig. 8D,E,O). Furthermore, knockdown of *MAP2K5* rescued motor axonopathy induced by overexpression of the mutant ANG variants (Fig. 8L–O), whereas injection of control MO did not (Fig. 8I–K,O). Taken together, these data indicate that MAP2K5 has a disease modifying function in the mutant OPTN- and ANG-induced zebrafish models of ALS. Expression of *MAP2K5* in the zebrafish spinal cord was confirmed through in situ hybridization in zebrafish spinal cord and RT-PCR using FACS-sorted neurons (Supplemental Fig. S11). Immunofluorescence staining showed that OPTN, ANG, and MAP2K5/MAPK7 were expressed in the mouse spinal cord, suggesting a genetic interaction among these genes in the mammalian spinal cord and an important role for MAP2K5 in ALS patients (Supplemental Fig. S12). Because autophagy has been implicated in the pathogenesis of ALS (Chen et al. 2012; Nixon 2013; Monahan et al. 2016; Weishaupt et al. 2016), the role of MAP2K5 in autophagy was examined. Our results showed that MAP2K5 inhibition enhanced autophagy, as determined by LC3 cleavage and SQSTM1 degradation in NIH3T3 cells (Supplemental Fig. S13), as well as differentiated NSC-34 motoneuron-like cells (Supplemental Fig. S14). MAP2K5 inhibition increased autophagy flux (Supplemental Fig. S13B), which was evaluated from the fusion of autophagosomes and lysosomes as described previously (Klionsky et al. 2012; Nyfeler et al. 2012). These results suggest that *MAP2K5* knockdown in zebrafish augments the autophagy process to alleviate motoneuron damage. Deletion of MAP2K5 (*MKK1*) also enhanced autophagy in yeast (Supplemental Fig. S15), indicating that MAP2K5 regulates autophagy in both mammalian cells and yeast. The results also led us to speculate that knockdown or deletion of *MAP2K5* exerts protective effects against the ANG and OPTN toxicity in zebrafish and yeast via similar mechanisms.

## Discussion

To enhance our understanding of disease pathways, a large-scale human–yeast genetic interaction screen was performed. Of 1305 human disease genes in the OMIM database, 20 genes were highly toxic when overexpressed in yeast (Fig. 1A; Table 1). OMIM gene-induced yeast toxicity formed the phenotypic basis for a genome-wide screen of human–yeast genetic interaction (Fig. 2). En masse transformation of the toxic OMIM genes into a barcoded yeast deletion library and subsequent multiplexed barcode sequencing identified OMIM-yeast genetic interactions. Genetic interactions between human disease genes and yeast genes have been investigated previously (Giorgini et al. 2005; Cooper et al. 2006; Gitler 2008). However, in the previous studies, a single human gene was introduced into each yeast deletion strain in an array format. Compared with the method used in the previous studies, our method is faster and more cost effective; it identified genome-wide human–yeast genetic interactions for 20 OMIM genes at the same time in a pooled and multiplexed format. However, our method has a few drawbacks. Because genetic interaction in the current method is based solely on the suppression of OMIM gene toxicity in yeast deletion strains, disease genes that are not toxic in yeast under any growth conditions will not be suitable for this screen. Although this method did not efficiently identify genetic interactions that enhanced OMIM toxicity, we expect that it could be achieved by variants of the approach described here (e.g., by using a lower expression level of the human gene to reduce baseline toxicity or via deeper barcode sequencing at earlier time points to more quantitatively detect underrepresentation of specific deletion strains). It should also be noted that the toxicity phenotype was used as a starting point to analyze genetic interactions allowing us to know more about the biology of these proteins. Overexpression in yeast might not be the best approach, because the disease mechanisms might be related to loss of function for recessive mutations.

OMIM gene toxicity has been linked to the formation of protein aggregates in yeast. This feature was used to analyze the genetic interactions of the ALS-associated genes. Genes involved in neurodegenerative diseases often form protein aggregates when expressed in yeast, leading to cellular toxicity (Giorgini et al. 2005; Cooper et al. 2006; Johnson et al. 2008). Of the 20 OMIM genes screened, *OPTN* and *ANG*, which have been linked to ALS in previous reports, were the focus of further studies. In this study, OPTN and ANG overexpression similarly induced a protein aggregation phenotype (Fig. 1B). Consistently, OPTN has been shown to form toxic aggregates in yeast (Kryndushkin et al. 2012). Our results showed that cytoplasmic aggregates of OPTN and ANG were reduced in specific yeast deletion strains. The aggregation suppressors for OPTN and ANG were enriched for several biological functions, such as cell death, lipid metabolism, and trafficking (Figs. 4, 5; Tables 2, 3). These results suggest that lipid metabolism is centrally involved in ALS pathogenesis. Indeed, ceramide, cholesterol, and sphingomyelin have been shown to play important roles in ischemic stroke (Yu et al. 2000), Parkinson's disease (Hunot et al. 1997), and Alzheimer's disease (Cutler et al. 2004). Moreover, lipid accumulation mediates the oxidative stress-induced death of motoneurons in ALS patients and in an ALS animal model (Cutler et al. 2002; Simpson et al. 2004).

Overexpression of OPTN, ANG, and their disease-linked variants also induced protein aggregation in mammalian cells. Our subsequent studies focused on MAP2K5 (human ortholog to MKK1), one of the aggregation suppressors in yeast. Inhibition of MAP2K5 attenuated the formation of protein aggregates in NIH3T3 mouse fibroblast cells, indicating a genetic interaction between OPTN/ANG and MAP2K5 in mammalian cells. Zebrafish studies showed that *MAP2K5* knockdown partly rescued motoneuron degeneration caused by ectopic expression of OPTN, ANG, or their variants, indicating a genetic interaction occurs in vivo. MAP2K5 is an upstream regulator of MAPK7. MAP2K5/MAPK7 pathways have been implicated in diverse cellular processes such as cell survival, apoptosis, motility, differentiation, and proliferation (Drew et al. 2012). Because of the involvement of MAP2K5/MAPK7 in angiogenesis, the epithelial-mesenchymal transition, and diverse oncogenic pathways, the role of MAP2K5/MAPK7 in cancer has been the subject of intense investigation for the last decade (Lochhead et al. 2012). In the nervous system, MAP2K/MAPK pathways are associated with neuronal cell death (Subramaniam et al. 2004; Subramaniam and Unsicker 2010) and survival (Wang et al. 2005; Cavanaugh et al. 2006; Parmar et al. 2014), as well as with neuropathic pain (Ma and Quirion 2005). In particular, MAPK7 has been implicated in neuronal survival and death (Cavanaugh 2004; Subramaniam et al. 2004). Overexpression of MAPK7 has been shown to promote apoptotic cell death in medulloblastoma cell lines (Sturla et al. 2005). MAPK7 suppresses brain-derived neurotrophic factor (BDNF) expression in the glial cells (Su et al. 2011). MAPK7 also plays a critical role in the BDNF-promoted survival of developing, but not mature, cortical neurons (Liu et al. 2003). How MAP2K5/MAPK7 participates in neuronal cell death/survival paradigms remains to be determined. OPTN mutations have been shown to affect regulation of NF-κB signaling (Zhu et al. 2007; Nagabhushana et al. 2011). MAP2K5 inhibition, however, did not significantly affect these functions of OPTN (Supplemental Fig. S16). Together, our results in yeast, mammalian cells, and zebrafish indicate that pharmacological or genetic inhibition of MAP2K5 reduces protein aggregation and thereby provides neuroprotection. Thus, our findings suggest that MAP2K5/MAPK7 pathways influence degeneration of motoneurons in ALS.

In summary, a genome-wide genetic screen for interactions between human disease genes and yeast genes provided a “first-draft” disease interactome, serving as the basis for future investigations of disease pathways. Our studies focusing on ALS-linked genes newly identified MAP2K5 as a promising drug target for the treatment of ALS. Other genes identified in the screen are potential therapeutic targets that deserve further investigation.

## Methods

### Yeast strains, media, and plasmids

BY4742 (Mat α; *his3Δ1*; *leu2Δ0*, *lys2Δ0*; *ura3Δ0*) was used as a wild-type yeast strain in this study. The Homozygous Diploid Complete Set of Yeast Deletion Clones and Homozygous Diploid Yeast Deletion Pools were purchased from Invitrogen. Yeast cells were grown in rich medium (YPD) or in synthetic medium lacking leucine and containing 2% glucose (SD-Leu), raffinose (SRaf-Leu), or galactose (SGal-Leu). Gateway entry clones of OMIM ORFs were obtained from the hORFeome V8.1 entry clone collection (http://horfdb.dfci.harvard.edu). All entry clones contain full-length human OMIM ORFs without a stop codon. The Gateway LR reaction was used to shuttle OMIM ORFs into pAG425GAL-ccdB or pAG425GAL-ccdB-GFP (Addgene) (Alberti et al. 2007) for yeast expression. All plasmids are 2-µm based and under the control of the *GAL1* promoter. All constructs were verified by Sanger sequencing. For functional studies in mammalian cells or zebrafish, the Gateway LR reaction was used to shuttle OMIM ORFs into the pDS-GFP-XB (Invitrogen) or pCSDest (Addgene) destination vectors. Human OPTN or ANG variants were generated with the QuikChange Site-Directed Mutagenesis Kit (Stratagene), as described in the manual, using specific oligonucleotides.

### Yeast transformation and spotting assays

OMIM ORFs in pAG425GAL (yeast destination vector) were transformed into BY4742 or homozygous diploid deletion strains. All yeast strains were grown at 30°C according to the standard protocol. We used the LiAc/SS carrier DNA/PEG method to transform yeast with plasmid DNA as previously described (Gietz and Schiestl 2007). For spotting assays, yeast cells were grown overnight at 30°C in SRaf-Leu media. Cultures were serially diluted and spotted onto SD-Leu or SGal-Leu medium and grown for 2–4 d at 30°C.

### Human–yeast genetic interaction screen

OMIM ORFs were transformed into homozygous diploid yeast deletion pools containing 4653 individual deletion clones. Transformants were selected by incubating cells in 5 mL of SD-Leu media. To determine the transformation efficiency, 0.1% of the cells (5 µL) were plated onto SD-Leu agar plates. Approximately 50–100 individual transformants were obtained, indicating 10- to 20-fold coverage of the deletion library. Transformants were incubated in SD-Leu medium for 16 h. The cells were washed twice with PBS and then incubated in SGal-Leu medium for 2 d. Genomic DNA was isolated from cells harvested at the end of pooled growth. Each 20-mer uptag barcode was amplified using composite primers comprised of the sequences of the indexing tag and the sequences of the common barcode primers: 5′-G*NNNNNN***GATGTCCACGAGGTCTCT**-3′ (forward) and 5′-C*NNNNNN***GTCGACCTGCAGCGTACG**-3′ (reverse). The 5′ portion (italics) is the variable sequence, which represents the 6-mer indexing tag used for multiplexing. The 3′ portion (bold) represents the common primer flanking the uptag barcode; it is required to amplify the yeast barcodes. PCR amplification was carried out at an annealing temperature of 55°C for 30 cycles using a DNA Engine Tetrad Peltier Thermal Cycler (MJ Research). The PCR products were gel-purified from 4% agarose gels. Equal volumes of normalized DNA were then pooled in one tube and sequenced using a Genome Analyzer (Illumina) according to the manufacturer's protocols.

### Analysis of Illumina sequencing

To analyze the barcode sequencing (Bar-seq) data, all counts were quantile normalized such that each barcode number was divided by the average of the barcode numbers. This ensured that each OMIM experiment had the same count distribution. The normalized barcode number was converted into a *Z*-score for each yeast gene. The error rate for the *Z*-score was also calculated by error propagation, starting from the estimated error rate of the barcode number. The corrected *Z*-score was calculated using the error rate as follows: corrected *Z*-score = (1 ± error rate) × Z-score.

### Bioinformatic analysis and network construction

IPA (http://analysis.ingenuity.com) and DAVID (http://david.abcc.ncifcrf.gov/) were used to construct networks of protein–protein interaction, transcriptional regulation, phosphorylation, subcellular localization, and molecular function.

### Fluorescence detection of protein aggregates in yeast

Yeast cells were transformed with plasmids encoding GFP-fused OMIM proteins. The expression of GFP-fused OMIM proteins was induced by incubating cells in SGal-Leu for 16 h. The yeast cells were then fixed in 4% paraformaldehyde for 30 min. The fixed cells were placed on cover slides and observed under a fluorescence microscope (Olympus BX51) attached to a CCD color video camera (Olympus D70).

### Cell cultures and detection of protein aggregates

NIH3T3 mouse fibroblasts and differentiated NSC-34 mouse motoneuron-like cells were grown and maintained in Dulbecco's modified Eagle's medium (DMEM) containing 10% fetal bovine serum, 2 mM glutamine, penicillin, and streptomycin (Gibco). NIH3T3 cells were seeded at a density of 5 × 10^4^ cells per well in 24-well plates and transfected with GFP-fused OPTN or ANG. At 48 h after transfection, cells were fixed with 4% paraformaldehyde for 30 min. Cells were observed with fluorescence microscopy (Olympus BX51). Soluble and insoluble OPTN and ANG proteins were detected as described previously (Korac et al. 2013; Minegishi et al. 2013). In brief, NIH3T3 cells were seeded at a density of 5 × 10^4^ cells per well in 24-well plates and transfected with various plasmids in the presence or absence of the MAP2K5 inhibitor BIX 02189 (Selleckchem). DMSO (0.1% v/v) was used as a vehicle. For protein fractionation, cells were lysed in NP40 lysis buffer (50 mM Tris, 150 mM NaCl, 1 mM EDTA, 5 mM MgCl_2_, 0.5% NP40) supplemented with Complete Protease Inhibitor Cocktail (Roche). After centrifugation for 10 min at 12,000 rpm, the supernatant, which constituted the soluble fraction, was collected. The pellet was resuspended in NP40 buffer containing 2% SDS to yield the insoluble fraction. The soluble and insoluble fractions were analyzed with SDS-PAGE followed by Western blot detection of GFP-fused proteins using an anti-GFP antibody (Santa Cruz Biotechnology; sc-9996). For some experiments, NIH3T3 cells were transfected with the mCherry-GFP-LC3 plasmid to monitor autophagy flux, as described previously (Klionsky et al. 2012; Nyfeler et al. 2012).

### Western blot analysis

For the detection of autophagy, protein extracts of NIH3T3 cells after treatment with BIX 02189 or rapamycin (Sigma) were separated by SDS-PAGE, blotted, and incubated with a polyclonal anti-LC3 antibody (MBL International; M152-3) or anti-p62 antibody (Enzo Life Sciences; BML-PW9860). Incubation with secondary antibodies and chemiluminescence detection followed. MAPK7/phospho-MAPK7, beta actin, and alpha tubulin were similarly detected using an anti-MAPK7 antibody (Cell Signaling; #3372), an anti-phospho-MAPK7 antibody (Cell Signaling; #3371), an anti-beta actin antibody (Invitrogen; MA5-15739), and an anti-alpha tubulin antibody (Sigma; T5168), respectively. For ATG8 lipidation immunoblot analysis in yeast, wild-type and ΔMKK1 yeast cells were grown to an OD_600_ of 1 in SGal-Leu medium and harvested. For starvation control, cells were washed twice with SD(−N) medium and incubated for 6 h. Samples were collected and subjected to immunoblot analysis using antibodies against ATG8 (Abcam; ab4753). Separation of ATG8 from ATG8–PE (phosphatidylethanolamine) was done by adding 6 M urea to standard 13.5% SDS-polyacrylamide gels as described previously (Kirisako et al. 2000).

### Immunofluorescence analysis

Specific proteins were detected in mammalian cells and mouse tissues using immunofluorescence analysis as previously described (Nam et al. 2014).

### Screening in a zebrafish system

All animal experiments were carried out in accordance with the guidelines in the NIH Guide for the Care and Use of Laboratory Animals. The experiments were approved by an institutional review board.

#### Zebrafish lines

*Tg(olig2:dsred2)* (Kucenas et al. 2008) and *Tg(huC:egfp)* (Park et al. 2000b) zebrafish of either sex were used for this study. To block pigmentation in zebrafish, 0.003% (w/v) 1-phenyl-2-thiourea (PTU) was added to the embryo medium at 24 h post fertilization.

#### Morpholino and RNA injection

For morpholino-based knockdown of MAP2K5, we designed a translation-blocking morpholino oligonucleotide (MO) against *map2k5* (*map2k5* MO): control MO (5′-CCTCTTACCTCAGTTACAATTTATA-3′) and *map2k5* MO (5′-ACACACCGACAAACATAATCTTGGC-3′). The oligos were synthesized by Gene Tools. The MOs were dissolved in 1× Danieau's solution at a concentration of 20 µg/µL and diluted further with distilled water. OPTN and ANG WT and mutant mRNAs were produced using the mMESSAGE mMACHINE RNA Synthesis Kit (Ambion) and purified with a MEGAclear Kit (Ambion). Purified mRNAs were diluted to a final concentration of 30 ng/µL, and 300 pg of mRNA was injected into one-cell-stage zebrafish embryos with 5 ng of MOs.

#### Plasmid construction

To produce the *cmv:map2k5-egfp* and *hsp70:map2k5-mCherry* constructs, zebrafish *map2k5* (GenBank Accession No. EF433292) was amplified by PCR using primers containing *att*B1 and *att*B2 sites (Supplemental Methods). The PCR product containing the attB sites was cloned into a middle-entry vector using the BP reaction of the Gateway system (Invitrogen). A 5′ entry clone containing a fragment of the cytomegalovirus (*cmv*) promoter or zebrafish heat shock protein 70 (*hsp70*) promoter and a 3′ entry clone containing *egfp* or *mCherry* were kindly provided by Dr. Chien (University of Utah) (Kwan et al. 2007). The Gateway LR reaction was performed using LR Clonase II with the entry clones, according to the manufacturer's recommendations (Invitrogen).

#### FACS and RT-PCR

Approximately 1000 *Tg(huC:egfp)* embryos at 2 d post fertilization (Park et al. 2000a) were used for the isolation of EGFP^+^ neurons with FACS. Cell dissociation and FACS were performed as previously described (Chung et al. 2011) using a FACSAria II (Becton Dickinson). Isolated cells were subsequently homogenized in TRIzol solution (Invitrogen) for the purification of total RNA. cDNA was synthesized using an ImProm-II Reverse Transcription system (Promega), and the specific oligonucleotide primers were used for RT-PCR.

#### Whole-mount in situ hybridization

Whole-mount in situ RNA hybridization was performed with sense and antisense RNA probes for zebrafish *map2k5* as described previously (Hauptmann and Gerster 2000). Photos were taken using a differential interference contrast microscope (Axioskop; Zeiss).

#### Image analysis

For high-magnification in vivo imaging, embryos were anesthetized with 0.03% tricaine (Sigma-Aldrich) and mounted in 0.8% low-melting agarose (SeaPlaque Agarose; Lonza) on glass-bottomed 35-mm dishes (MatTek). Fluorescence images were collected using a LSM510 laser scanning confocal microscope (Zeiss). To test the ability of *map2k5* MO to knock down *map2k5* expression, one-cell-stage zebrafish embryos were injected with control MO or *map2k5* MO (5 ng) together with *hsp70:map2k5:mcherry* DNA and then heat shocked at 24 hpf by incubating them at 39°C for 30 min. After heat-shock induction, fluorescence images were collected, and fluorescence intensity was measured by NIS-Elements and ROI statistics software (Nikon).

### Statistical analysis

All data are presented as the mean ± SD from three or more independent experiments, unless otherwise stated. Different treatments were compared with Student's *t*-test, one-way ANOVA with Dunnett's multiple comparisons test, or χ^2^ tests using the SPSS software (version 18.0; SPSS Inc.). Differences with a *P*-value less than 0.05 were considered statistically significant.

## Data access

The Bar-seq data in this study have been submitted to the NCBI Sequence Read Archive (SRA; https://www.ncbi.nlm.nih.gov/sra/) under accession number SRP107732.

## Supplementary Material

Supplemental Material
