# Supplementary material for: Yeast genetic interaction screen of human genes associated with amyotrophic lateral sclerosis: identification of MAP2K5 kinase as a potential drug target
Source: Genome Res. 2017 Sep;27(9):1487–500. doi: 10.1101/gr.211649.116 (PMC5580709; doi:10.1101/gr.211649.116)
Supplement: Supplemental Material [file supp_gr.211649.116_Supplemental_Methods.docx]

**Supplemental Methods**

**Plasmids**

Human OPTN or ANG variants were generated with the QuikChange site-directed mutagenesis kit (Stratagene, La Jolla, CA), as described in the manual, using the following oligonucleotides: OPTN E50K mutation forward primer, 5′-GAG CTC CTG ACC AAG AAC CAC CAG CTG AA-3′; E50K mutation reverse primer, 5′-CAG CTG GTG GTT CTT GGT CAG GAG CTC T-3′; OPTN E478G mutation forward primer, 5′-GAT TTT CAT GCT GGA AGA GCA GCG AGA G-3′; E478G mutation reverse primer, 5′-CTC GCT GCT CTT CCA GCA TGA AAA TCA G-3′; ANG K17I mutation forward primer, 5′-ACT ATG ATG CCA TAC CAC AGG GCC GGG A-3′; K17I mutation reverse primer, 5′-GGC CCT GTG GTA TGG CAT CAT AGT GCT-3′; ANG K40I mutant forward primer, 5′-GAC CTC ACC CTG CAT AGA CAT CAA CA-3′; ANG K40I mutant reverse primer, 5′-TGT GTT GAT GTC TAT GCA GGG TGA GG-3′; ANG P112L mutation forward primer, 5′-AAT GGC TTA CTT GTC CAC TTG GAT CAG-3′; and P112L mutation reverse primer, 5′-CCA AGT GGA CAA GTA AGC CAT TTT CAC-3′. To produce the *cmv:map2k5-egfp* and *hsp70:map2k5-mCherry* constructs, zebrafish *map2k5* (GenBank Accession No. EF433292) was amplified by PCR using a forward primer containing an attB1 site (5′-GGGGACAAGTTTGTACAAAAAAGCAGGCTTTAGCCAAGATTATGTTTGTCGGTGTGT-3′) and a reverse primer containing an attB2 site (5′- GGGGACCACTTTGTACAAGAAAGCTGGGTTGGACATGAGTCTCTGTGGCT-3′).

**Bioinformatic analysis and network construction**

After we identified the human orthologs of the yeast toxicity modifiers, the database accession numbers were imported into Ingenuity Pathways Analysis (IPA; Ingenuity Systems) for network analysis. The statistical significance of each network or list was determined with IPA using a Fisher Exact test (p < 0.05). IPA was also used to construct networks of protein-protein interaction, transcriptional regulation, and phosphorylation. Subcellular localization and molecular function were analyzed with DAVID (<http://david.abcc.ncifcrf.gov/>).

**RT-PCR**

Total RNA was extracted from NIH3T3 cells cultured in six-well plates using TRIzol reagent (Invitrogen) according to the manufacturer’s instructions. Reverse transcription was conducted using Superscript II (Invitrogen) and oligo(dT) primers. The following oligonucleotide primers were used for RT-PCR: *Ang* forward, 5′-GCCCGTTGT TCTTGATCTTC-3′; *Ang* reverse, 5′-ATCGAAGTGGACAGGCAAAC-3′; *Optn* forward, 5′-CGGAAGACTCCTTCGTTGAG-3’; *Optn* reverse, 5’-TTCAGCTCTGATGGTGTTGC-3’.

**Immunofluorescence analysis**

Specific proteins were detected in mammalian cells and mouse tissues using immunofluorescence analysis as previously described (Nam et al. 2014). Cells or tissue sections were sequentially incubated with primary antibodies [an anti-OPTN antibody (Abcam; ab23666), an anti-ANG antibody (Abcam; ab10600), an anti-MAPK7 antibody (Cell Signaling; #3372), an anti-MAP2K5 antibody (Santa Cruz Biotechnology; sc-135986) and an anti-NeuN antibody (Millipore, Billerica, MA; MAB377)] and fluorochrome-labeled secondary antibodies. Stained cells were examined under a fluorescence microscope.

**Zebrafish FACS and RT-PCR**

Approximately 1,000 *Tg(huC:egfp)* embryos at 2 days post fertilization (Park et al. 2000a) were used for the isolation of EGFP^+^ neurons with FACS. Cell dissociation and FACS were performed as previously described (Chung et al. 2011) using a FACSAria II (Becton Dickinson). Isolated cells were subsequently homogenized in TRIzol solution (Invitrogen) for the purification of total RNA. cDNA was synthesized using an ImProm-II reverse transcription system (Promega), and the following oligonucleotide primers were used for RT-PCR: *map2k5* forward, 5′-TGGAATCTGAGGGAATCGTC-3′; *map2k5* reverse, 5′-CTTACCGGCTCGTGGAAATA-3′; *huC* forward, 5′-CAAGGCTATCAACACGCTCA-3′; *huC* reverse, 5′-GCGCGATATACCTGTGACCT-3′; *egfp* forward, 5′-TATATCATGGCCGACAAGCA-3′; *egfp* reverse, 5′-GAACTCCAGCAGGACCATGT-3′; *flk* forward, 5′-TCGTGCTAGCAATGATGGAG-3′; *flk* reverse, 5′-CATCGAGAGGCATCTGT-3′; *beta-actin* forward, 5′-TAGTCATTCCAGAAGCGTTTACC-3′; and *beta-actin* reverse, 5′-TACAGAGACACCCTGGCTTACAT-3′.

**References**

Chung AY, Kim S, Kim H, Bae YK, Park HC. 2011. Microarray screening for genes involved in oligodendrocyte differentiation in the zebrafish CNS. *Exp Neurobiol* **20**: 85-91.

Nam Y, Kim JH, Seo M, Jin M, Jeon S, Seo JW, Lee WH, Bing SJ, Jee Y, Lee WK et al. 2014. Lipocalin-2 Protein Deficiency Ameliorates Experimental Autoimmune Encephalomyelitis: THE PATHOGENIC ROLE OF LIPOCALIN-2 IN THE CENTRAL NERVOUS SYSTEM AND PERIPHERAL LYMPHOID TISSUES. *J Biol Chem* **289**: 16773-16789.

Park HC, Hong SK, Kim HS, Kim SH, Yoon EJ, Kim CH, Miki N, Huh TL. 2000. Structural comparison of zebrafish Elav/Hu and their differential expressions during neurogenesis. *Neurosci Lett* **279**: 81-84.
