# Supplementary material for: Yeast genetic interaction screen of human genes associated with amyotrophic lateral sclerosis: identification of MAP2K5 kinase as a potential drug target
Source: Genome Res. 2017 Sep;27(9):1487–500. doi: 10.1101/gr.211649.116 (PMC5580709; doi:10.1101/gr.211649.116)
Supplement: Supplemental Material [file supp_gr.211649.116_Supplemental_Fig_S1.pdf]

Supplemental Figure 1

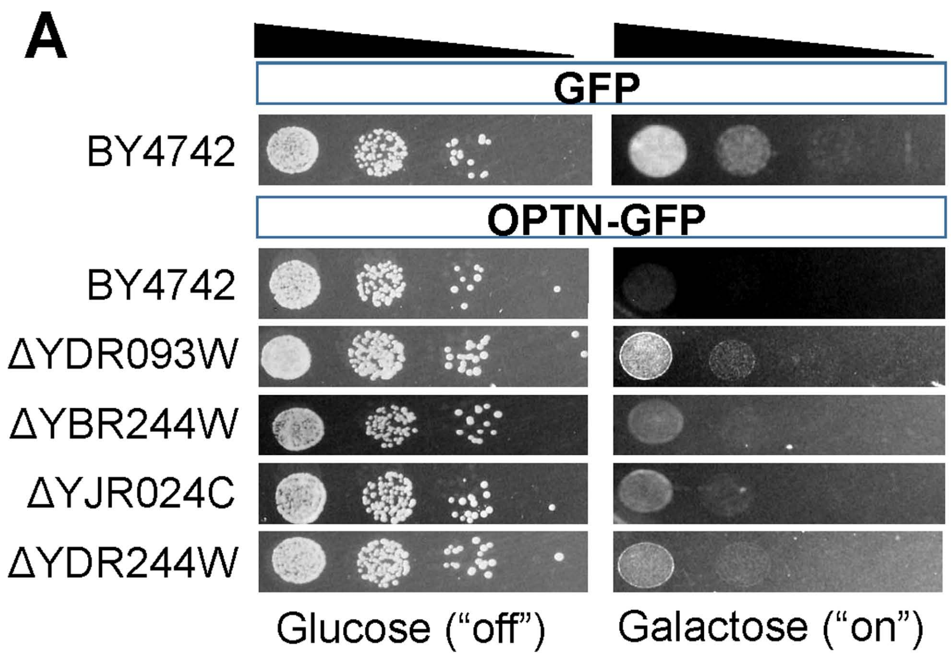

C

Systematic name	Standard name
YHR156C	LIN1
YPL064C	CWC27
YJR094C	IME1
YER176W	ECM32
YER072W	VTC1
YER053C	PIC2
YPL248C	GAL4
YOR009W	TIR4
YOR028C	CIN5
YAR023C	-
YHL042W	-
YOR007C	SGT2
YMR139W	RIM11
YDL204W	RTN2
YLR119W	SRN2
YDR093W	DNF2
YBR244W	GPX2
YJR024C	MDE1
YDR244W	PEX5

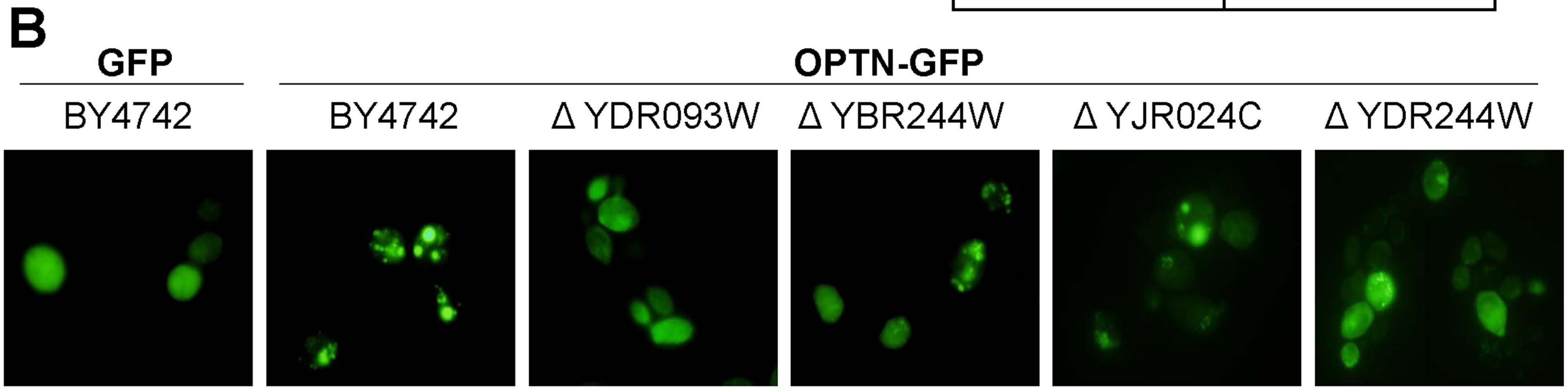

Supplemental Figure 1. Evaluation of yeast toxicity and protein aggregate formation after expression of GFP-fused OPTN. GFP-tagged OPTN induced yeast toxicity (*A*) and aggregate formation (*B*) in several yeast deletion strains in a manner similar to that of untagged OPTN protein. Systematic and standard names of the yeast deletion used here and Figure 3 are listed (*C*).
