# Supplementary material for: Yeast genetic interaction screen of human genes associated with amyotrophic lateral sclerosis: identification of MAP2K5 kinase as a potential drug target
Source: Genome Res. 2017 Sep;27(9):1487–500. doi: 10.1101/gr.211649.116 (PMC5580709; doi:10.1101/gr.211649.116)
Supplement: Supplemental Material [file supp_gr.211649.116_Supplemental_Fig_S2.pdf]

Supplemental Figure 2A

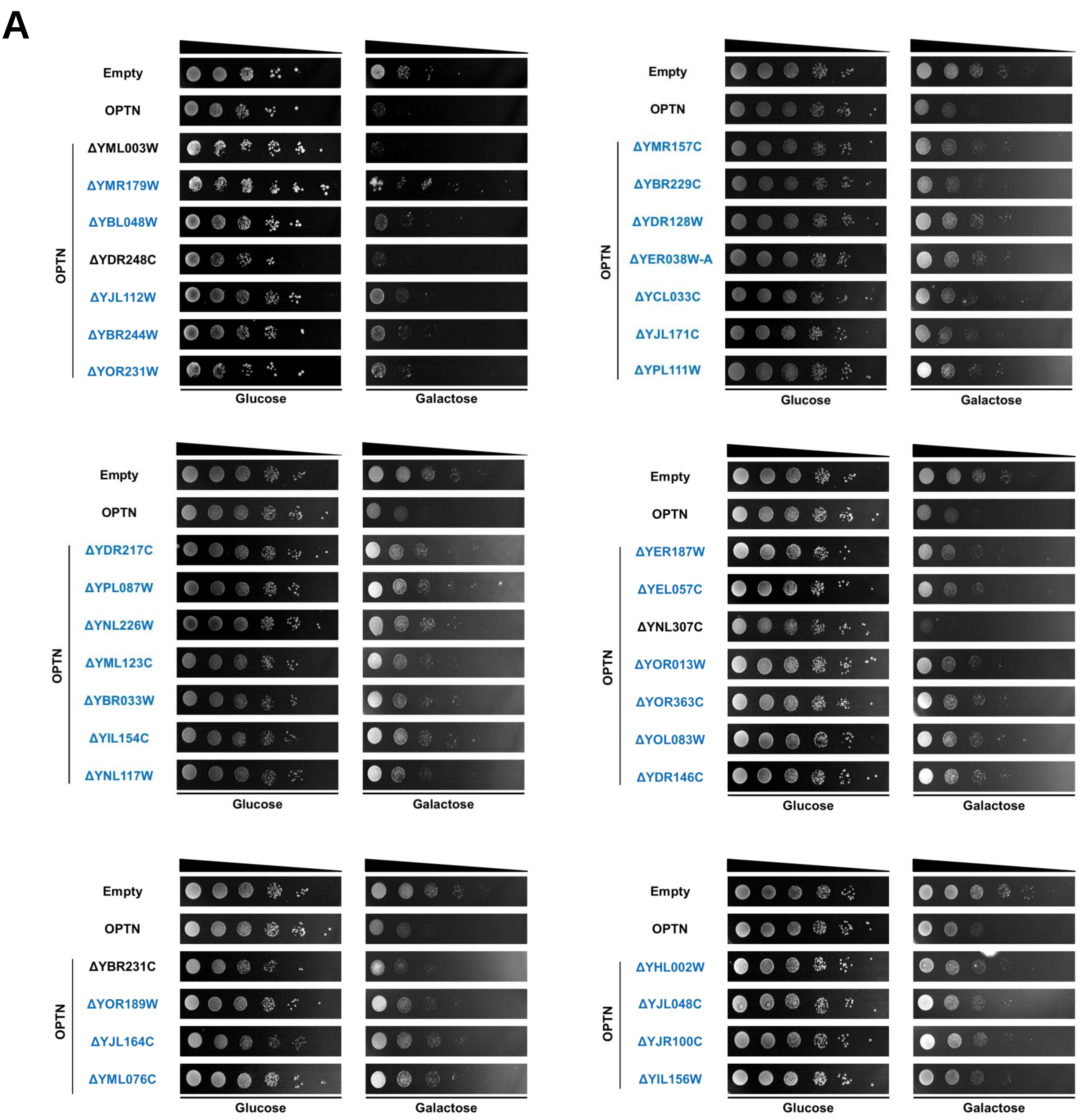

Supplemental Figure 2. Validation of toxicity modifiers by yeast spot assays. Ten percent of randomly selected modifying genes from toxicity suppressors and enhancers for the three OMIM genes (OPTN, ANG, and CLINT1) were tested by yeast spot assays. A, suppressors for OPTN; B, enhancers for OPTN; C, suppressors for ANG; D, enhancers for ANG; E, suppressors for CLINT1; F, enhancers for CLINT1; G, summary table. Deletion strains in blue indicate consistent results.

Supplemental Figure 2A continued

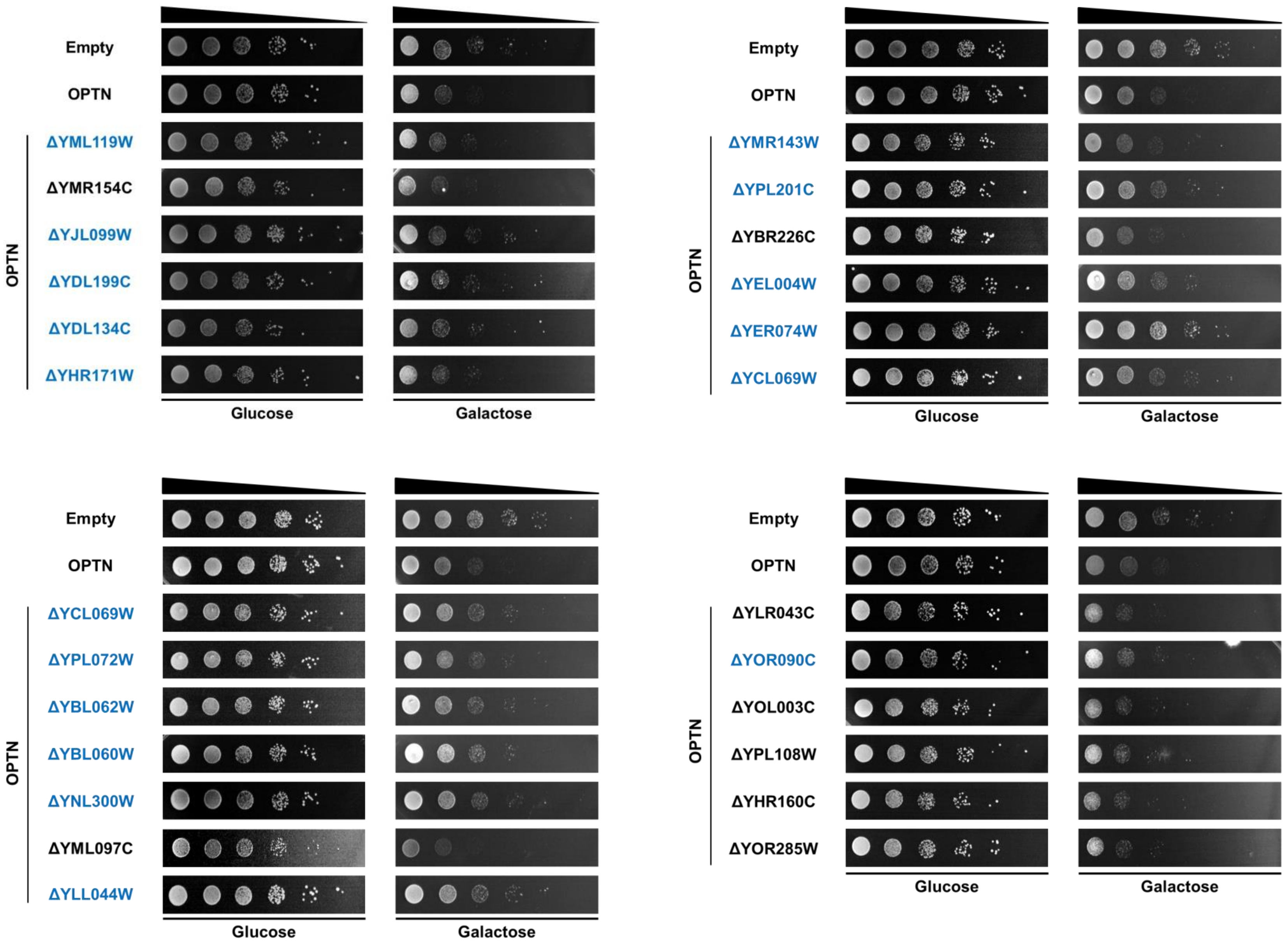

Supplemental Figure 2B

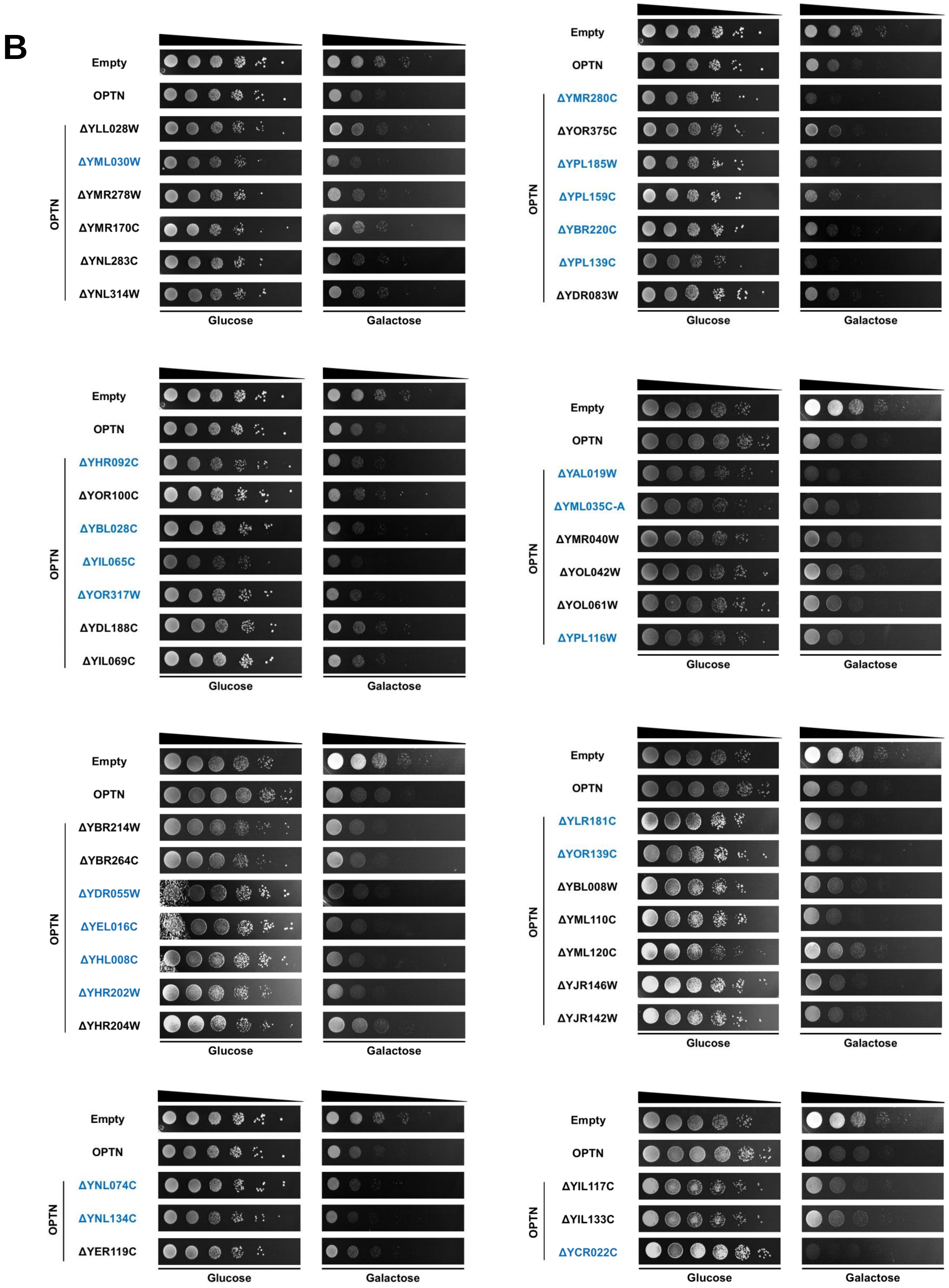

Supplemental Figure 2B continued

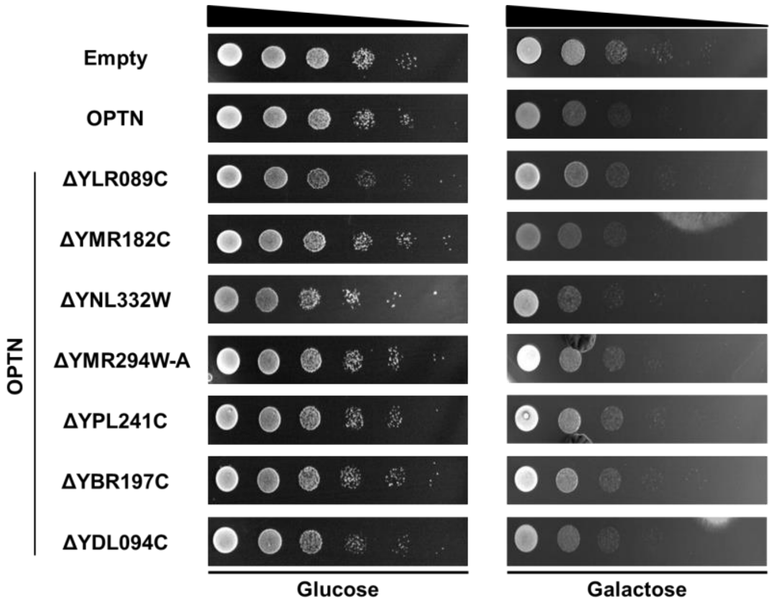

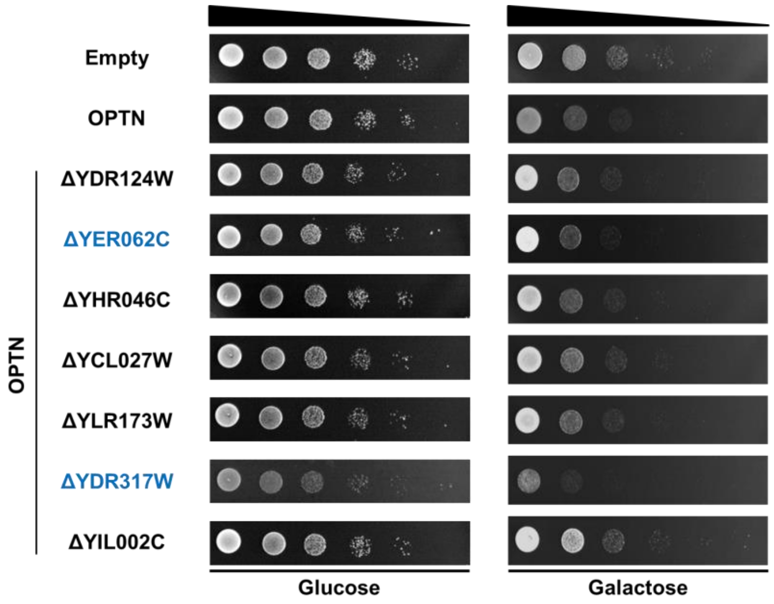

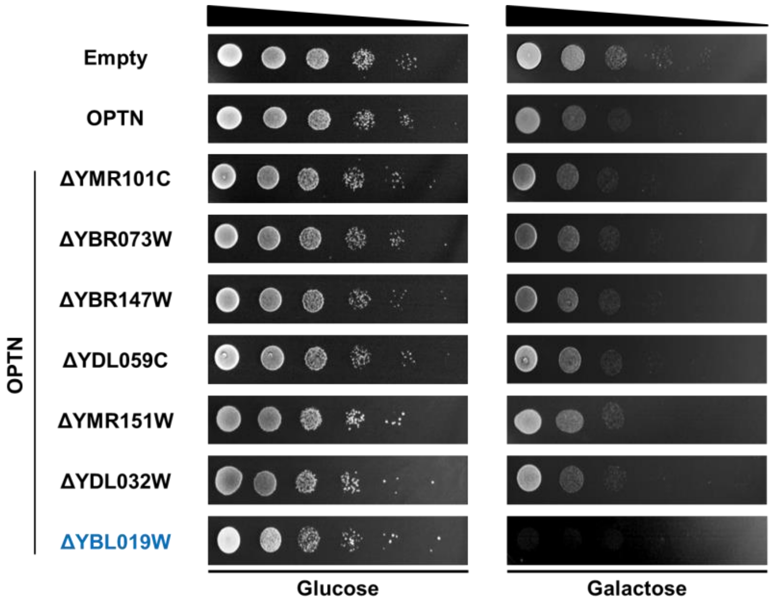

Supplemental Figure 2C

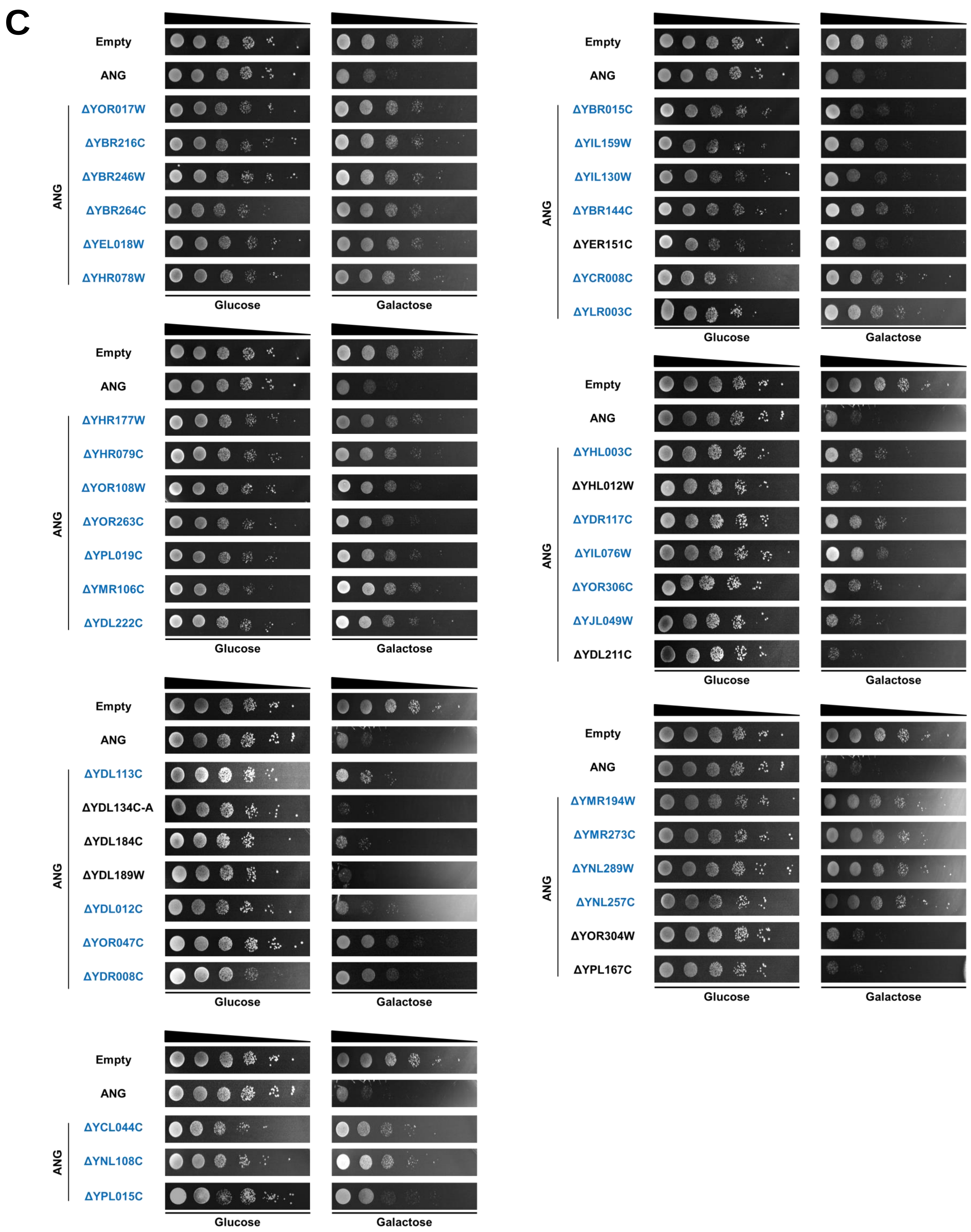

Supplemental Figure 2D

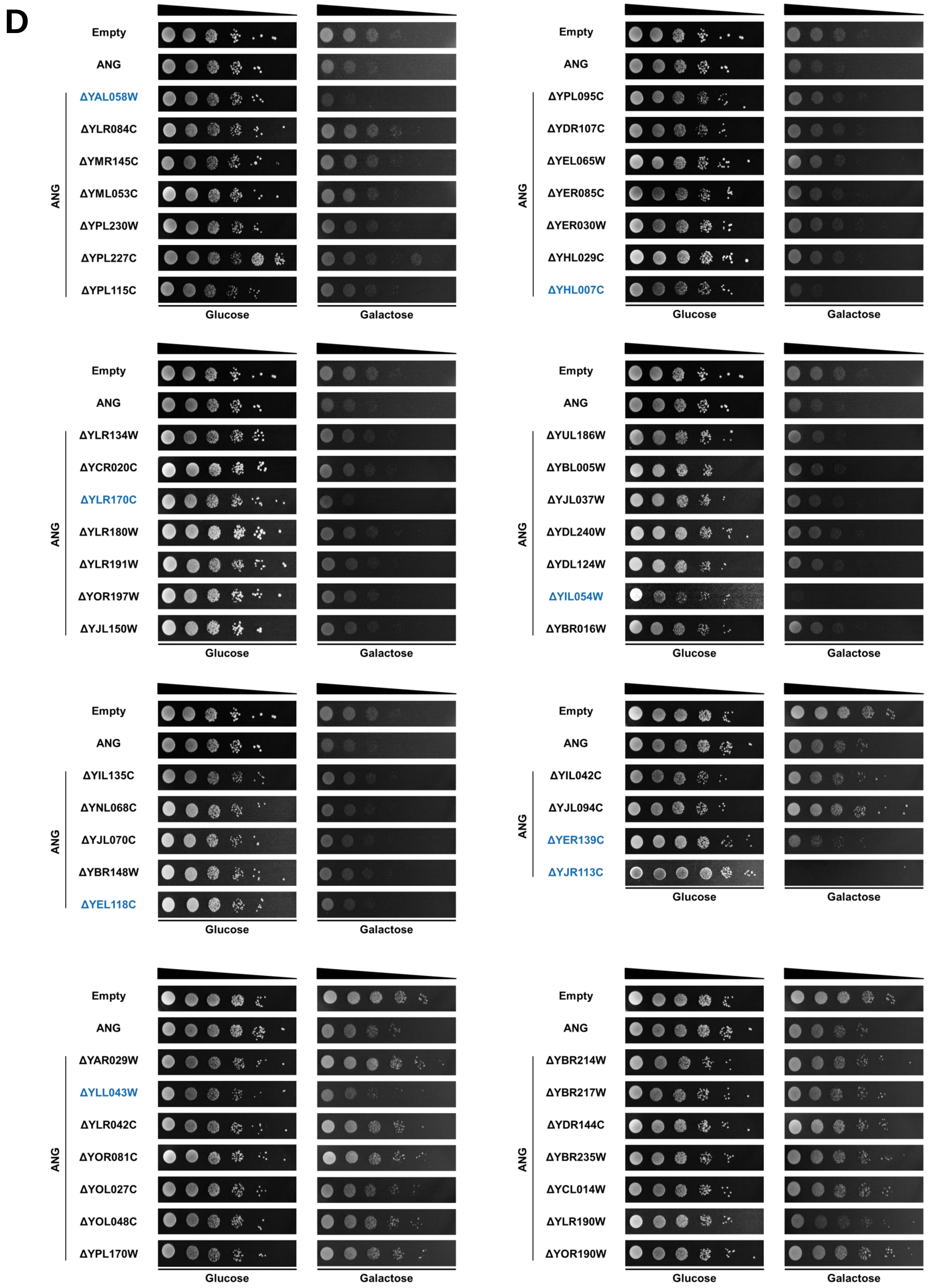

Supplemental Figure 2D continued

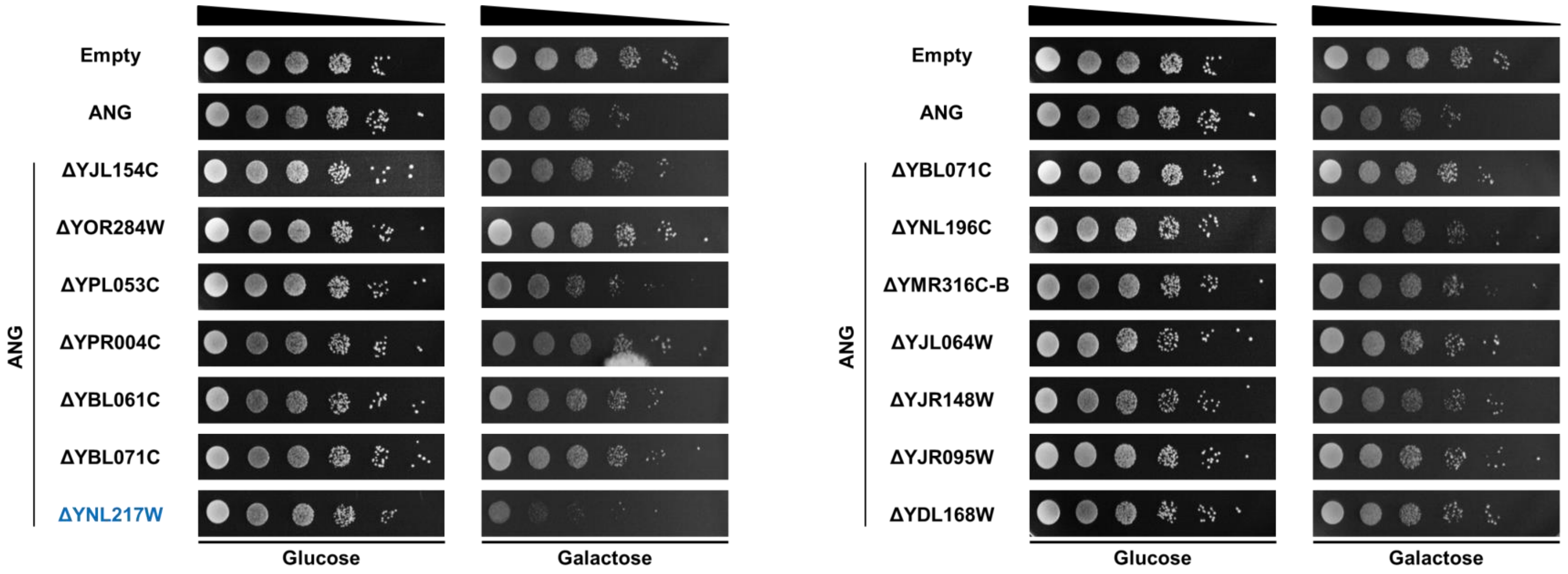

Supplemental Figure 2E

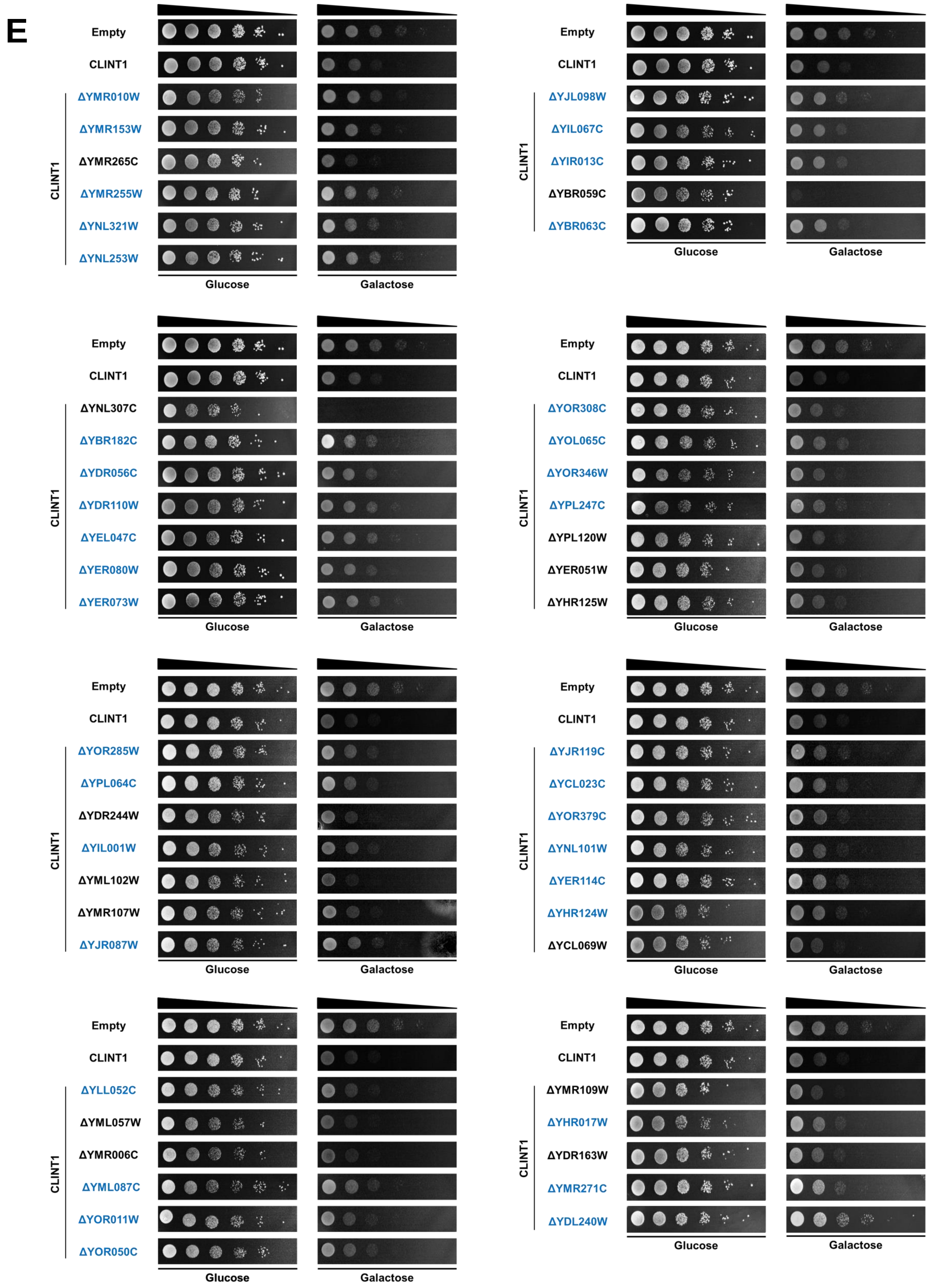

Supplemental Figure 2F

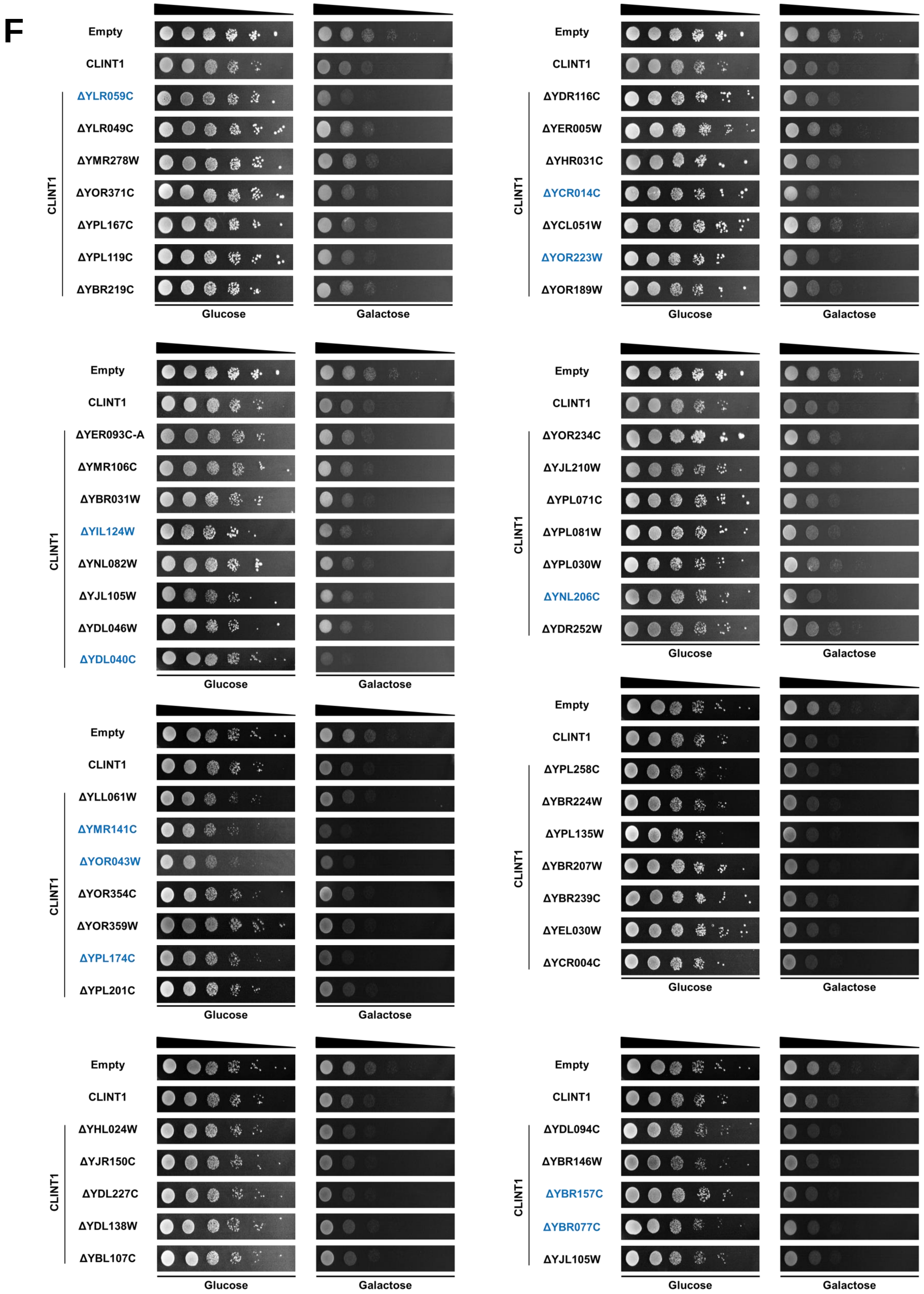

Supplemental Figure 2G

G

OMIM	Toxicity suppressors		Toxicity enhancers	
	No. suppressor/ No. tested	Consistency	No. enhancer/ No. tested	Consistency
OPTN	49/61	80.3%	25/67	37.3%
ANG	35/43	81.4%	8/65	12.3%
CLINT1	36/50	72.0%	14/59	23.7%
