# Supplementary material for: Yeast genetic interaction screen of human genes associated with amyotrophic lateral sclerosis: identification of MAP2K5 kinase as a potential drug target
Source: Genome Res. 2017 Sep;27(9):1487–500. doi: 10.1101/gr.211649.116 (PMC5580709; doi:10.1101/gr.211649.116)
Supplement: Supplemental Material [file supp_gr.211649.116_Supplemental_Fig_S3.pdf]

Supplemental Figure 3

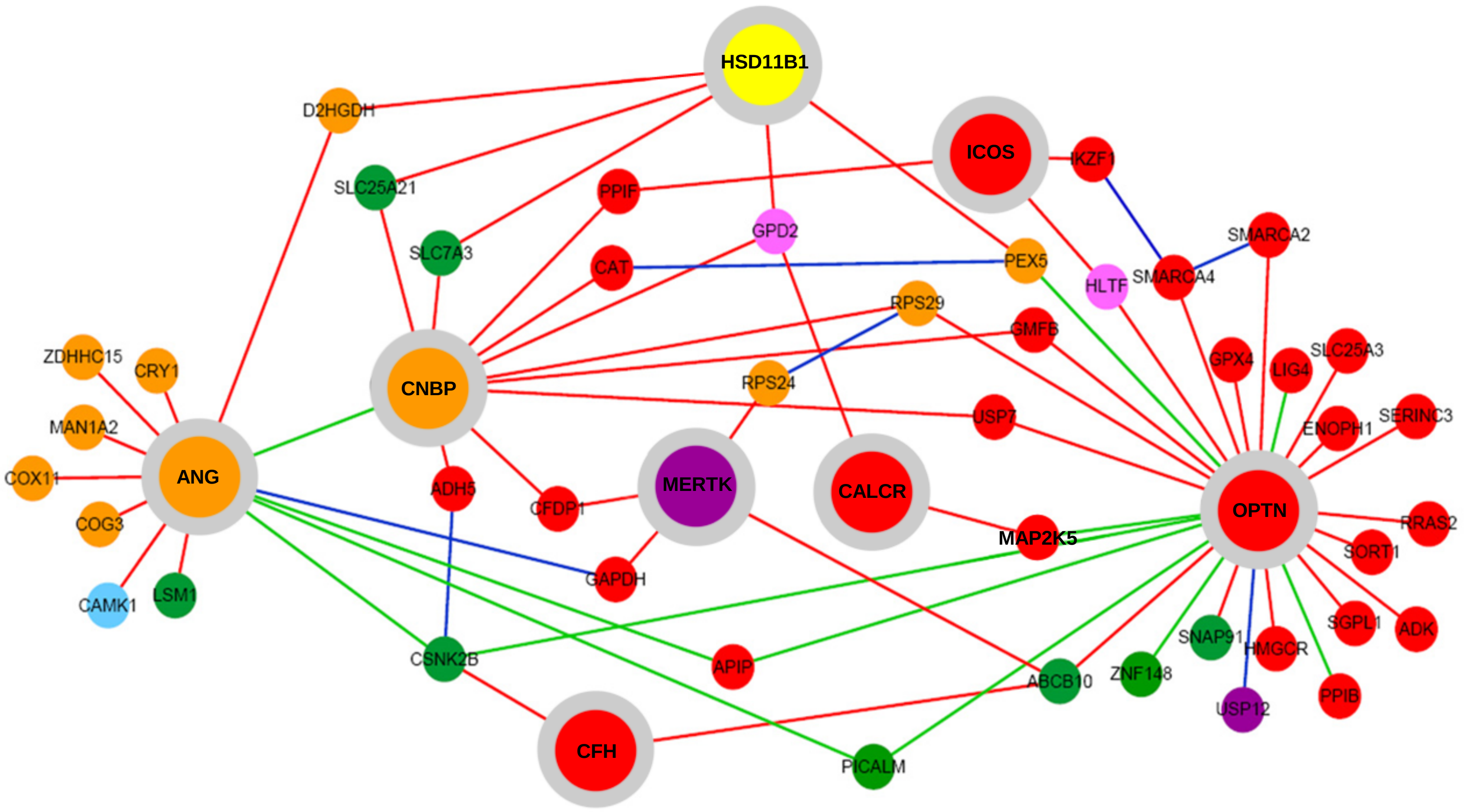

Node color: Cell death and survival; Protein synthesis; Lipid metabolism; Molecular transport; Post-translational modification; Cell cycle; Nucleic acid metabolism

Node with gray outline: OMIMs

Edge color: Predicted genetic interaction; Protein-protein interaction; Predicted and validated genetic interaction

Supplemental Figure 3. Subnetwork of human-yeast genetic interactions with a focus on OPTN and ANG. A subnetwork was constructed with OPTN/ANG and their toxicity modifiers with less than five interaction paths.
