# Supplementary material for: Yeast genetic interaction screen of human genes associated with amyotrophic lateral sclerosis: identification of MAP2K5 kinase as a potential drug target
Source: Genome Res. 2017 Sep;27(9):1487–500. doi: 10.1101/gr.211649.116 (PMC5580709; doi:10.1101/gr.211649.116)
Supplement: Supplemental Material [file supp_gr.211649.116_Supplemental_Fig_S5.pdf]

Supplemental Figure 5

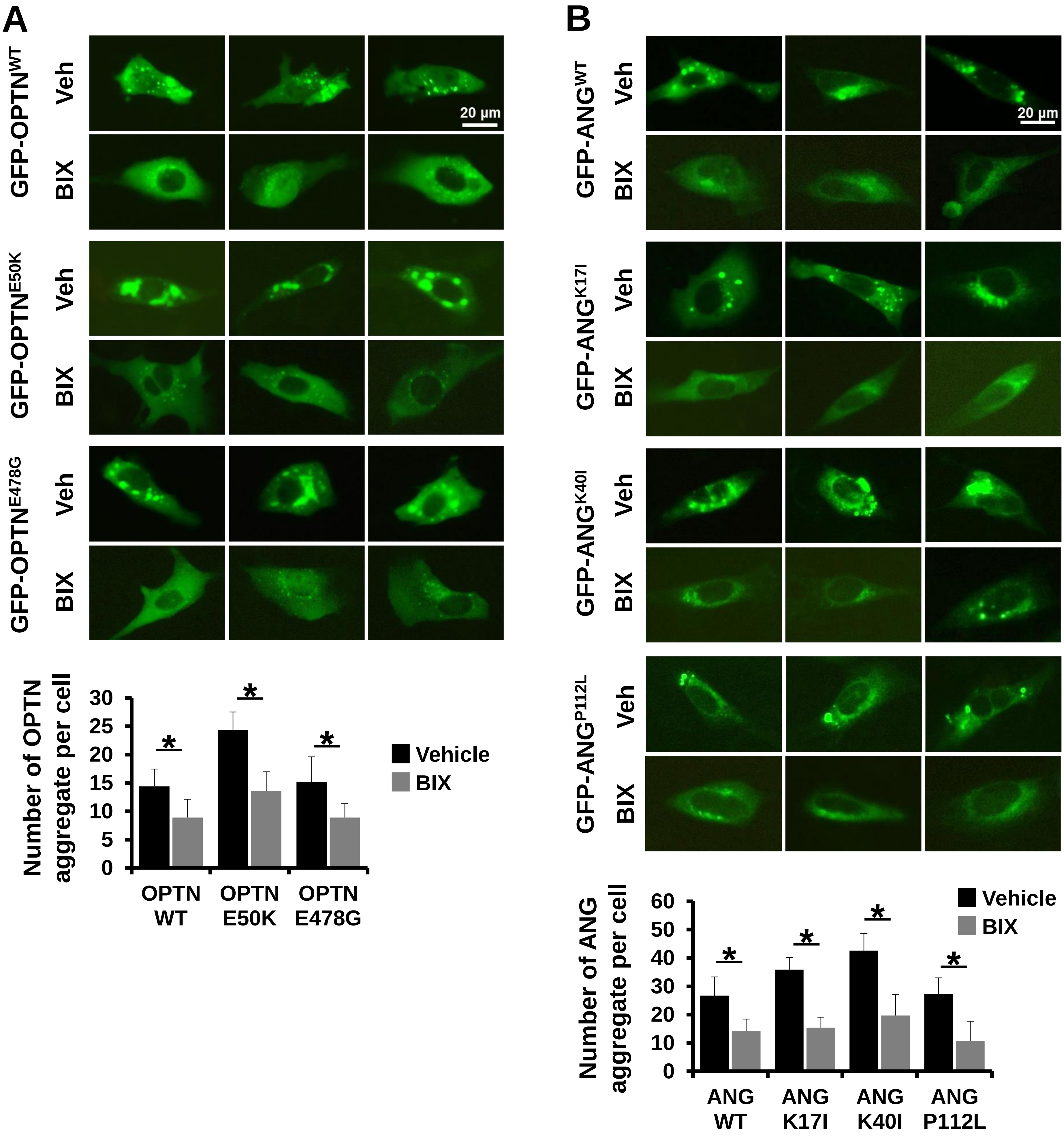

Supplemental Figure 5. Microscopy images of OPTN and ANG protein aggregates in mammalian cells. NIH3T3 cells were transiently transfected with wild-type GFP-OPTN or GFP-OPTN mutants (E50K or E478G) (*A*) and wild-type GFP-ANG or GFP-ANG mutants (K17I, K40I, or P112L) (*B*). After 36 hr of transient transfection, cells were incubated with vehicle (veh) or 10 µM BIX 02189 for 12 hr. Protein aggregates were visualized using fluorescence microscopy and quantified (*lower*). Protein aggregates (puncta (>1 µm)/cell) were counted using ImageJ software (Analyze particles). Results were obtained from at least 10 cells per group. Scale bar, 20 µm. *$p < 0.05$ between the indicated groups; mean ± SD.
