# Supplementary material for: Yeast genetic interaction screen of human genes associated with amyotrophic lateral sclerosis: identification of MAP2K5 kinase as a potential drug target
Source: Genome Res. 2017 Sep;27(9):1487–500. doi: 10.1101/gr.211649.116 (PMC5580709; doi:10.1101/gr.211649.116)
Supplement: Supplemental Material [file supp_gr.211649.116_Supplemental_Fig_S6.pdf]

**Supplemental Figure 6**

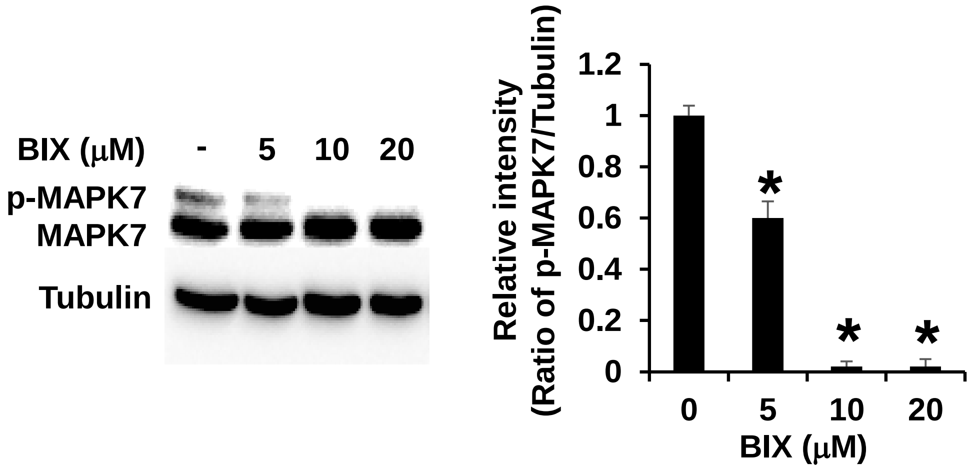


**Supplemental Figure 6. Inhibition of MAPK7 phosphorylation by BIX 02189.** NIH3T3 cells were incubated with increasing concentrations of BIX 02189 (0, 5, 10, 20 μM) for 2 hr. The cell lysates were harvested, and phosphorylated MAPK7 (p-MAPK7) was detected by western blot analysis. Inhibitor treatment reduced the basal levels of phospho-MAPK7. Tubulin was detected as a loading control. The results are representative of three experiments. The results of densitometric analysis (*right*) are presented as the mean ± SD ($n$ = 3); *$p < 0.05$ versus vehicle.
