# Supplementary material for: Yeast genetic interaction screen of human genes associated with amyotrophic lateral sclerosis: identification of MAP2K5 kinase as a potential drug target
Source: Genome Res. 2017 Sep;27(9):1487–500. doi: 10.1101/gr.211649.116 (PMC5580709; doi:10.1101/gr.211649.116)
Supplement: Supplemental Material [file supp_gr.211649.116_Supplemental_Fig_S7.pdf]

Supplemental Figure 7

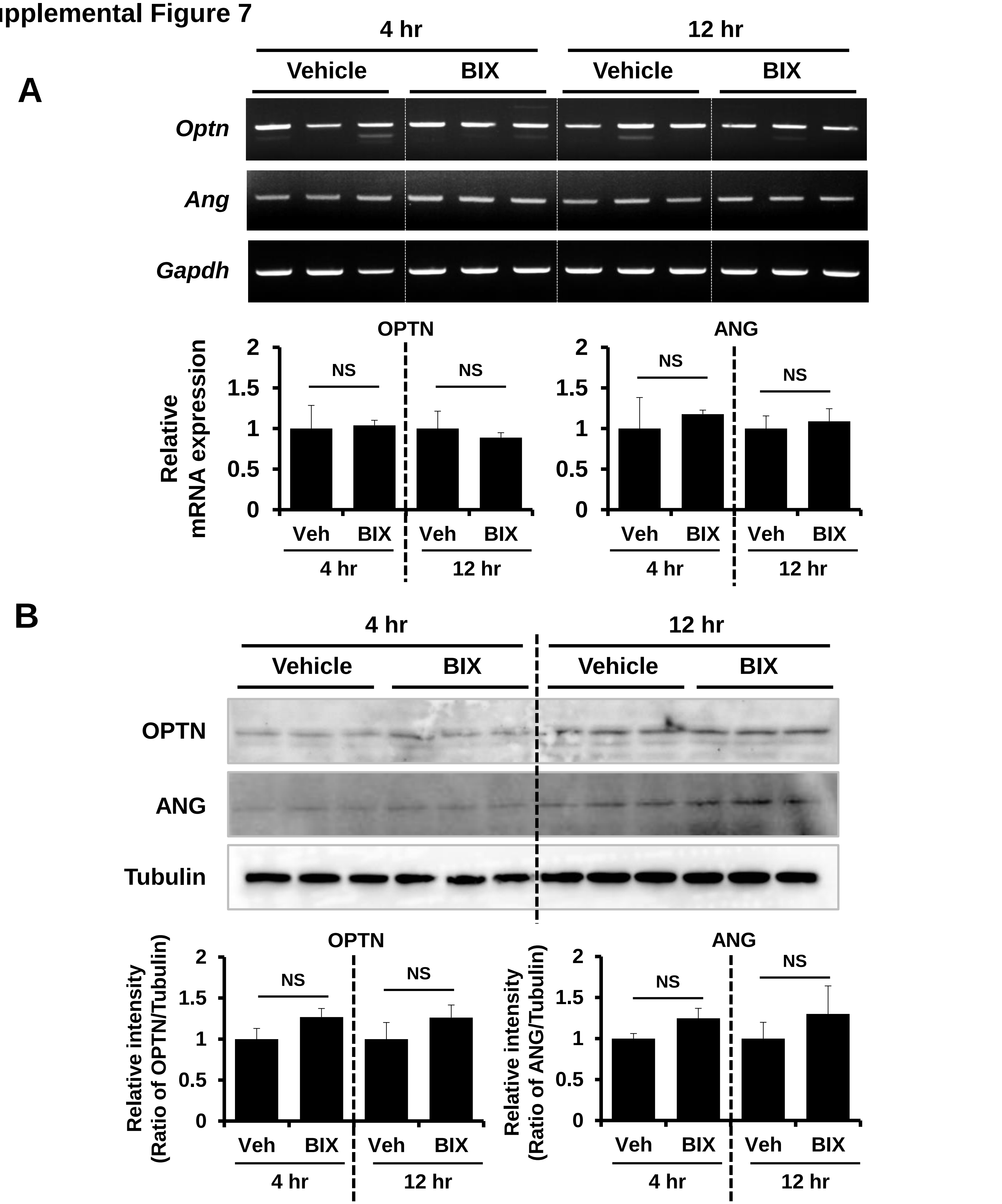

Supplemental Figure 7. No significant effects of MAP2K5 inhibition on OPTN or ANG levels. NIH3T3 cells were incubated with vehicle or BIX 02189 (10 μM) for 4 or 12 hr. The cell lysates were harvested, and the expression levels of OPTN and ANG mRNA (A) or protein (B) were assessed by RT-PCR (A) or western blot analysis (B), respectively. *Gapdh* and Tubulin were detected as a loading control. N.S. indicates no statistically significant difference (n=3).
