# Supplementary material for: Yeast genetic interaction screen of human genes associated with amyotrophic lateral sclerosis: identification of MAP2K5 kinase as a potential drug target
Source: Genome Res. 2017 Sep;27(9):1487–500. doi: 10.1101/gr.211649.116 (PMC5580709; doi:10.1101/gr.211649.116)
Supplement: Supplemental Material [file supp_gr.211649.116_Supplemental_Fig_S8.pdf]

Supplemental Figure 8

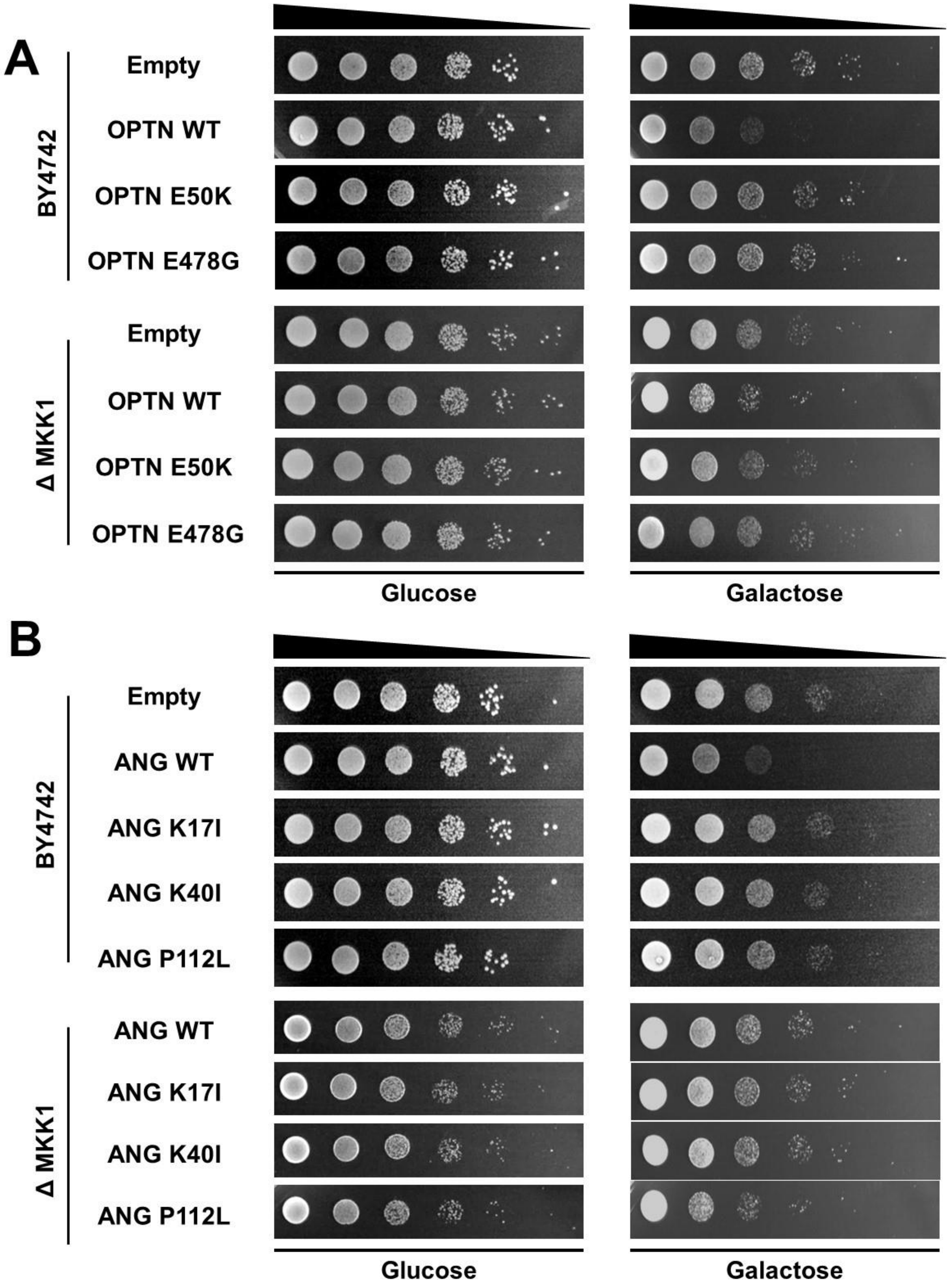

Supplemental Figure 8. Evaluation of WT/mutant OPTN/ANG genetic interactions in MKK1 deletion strain. WT and mutant OPTN and ANG toxicity in WT (BY4742) and MKK1 deletion strains was assessed by yeast spotting assay.
