# Supplementary material for: Yeast genetic interaction screen of human genes associated with amyotrophic lateral sclerosis: identification of MAP2K5 kinase as a potential drug target
Source: Genome Res. 2017 Sep;27(9):1487–500. doi: 10.1101/gr.211649.116 (PMC5580709; doi:10.1101/gr.211649.116)
Supplement: Supplemental Material [file supp_gr.211649.116_Supplemental_Fig_S9.pdf]

Supplemental Figure 9

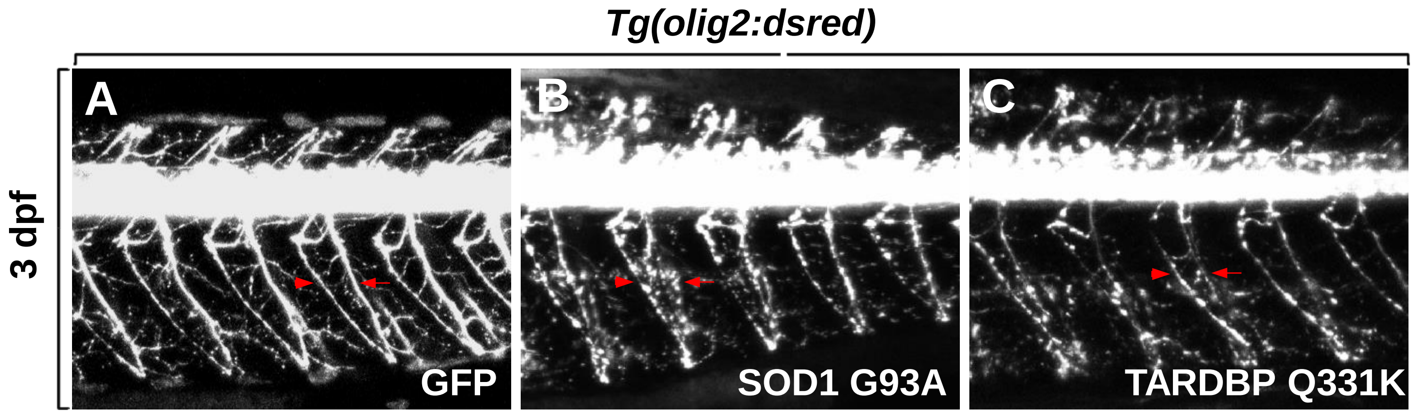

Supplemental Figure 9. Overexpression of SOD1 and TARDBP mutants causes motor axonopathy in the spinal cord of zebrafish embryo. All panels show lateral views of the spinal cord of *Tg*(*olig2:dsred2*) embryos, with anterior to the left and dorsal to the top. Motor axons (arrows) and neuromuscular junctions (NMJs, arrowheads) were detected with DsRed fluorescence protein expression. (A) Visualization of motor axons in the *egfp* mRNA-injected control embryos. (B, C) Injection of mRNA for SOD1 G93A (B) and TARDBP Q331K mutants (C) into the *Tg*(*olig2:dsred2*) embryos. Axonal defects are represented by axonal swelling and degeneration.
