# Supplementary material for: Yeast genetic interaction screen of human genes associated with amyotrophic lateral sclerosis: identification of MAP2K5 kinase as a potential drug target
Source: Genome Res. 2017 Sep;27(9):1487–500. doi: 10.1101/gr.211649.116 (PMC5580709; doi:10.1101/gr.211649.116)
Supplement: Supplemental Material [file supp_gr.211649.116_Supplemental_Fig_S11.pdf]

Supplemental Figure 11

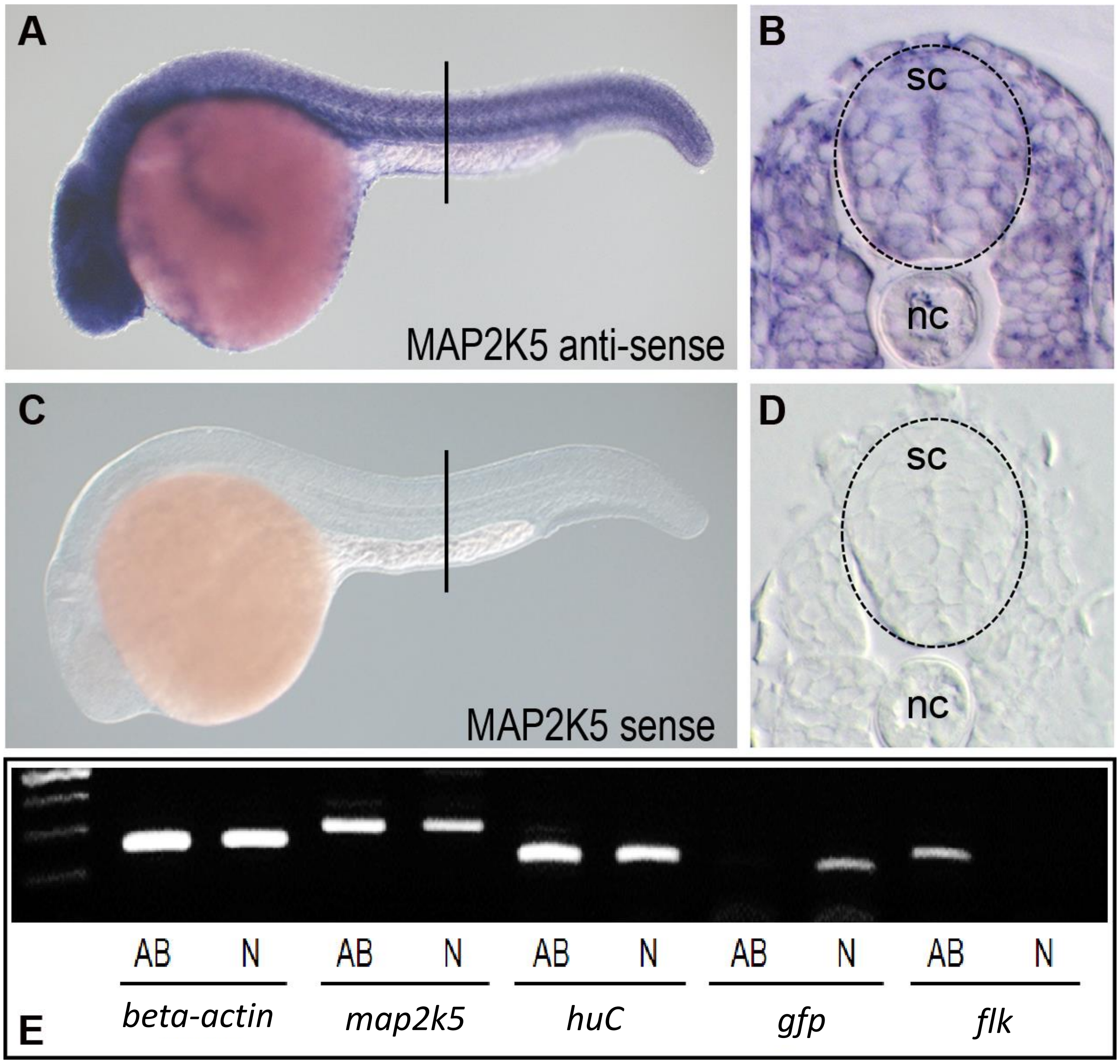

Supplemental Figure 11. Expression of MAP2K5 in developing zebrafish embryos. Whole-mount *in situ* RNA hybridization showed that *map2k5* was expressed ubiquitously in whole zebrafish embryos at 24 hr post fertilization (*A*). *map2k5* expression was also detected in whole spinal cord cells in a transverse section of the spinal cord, presumably including neurons (*B*), whereas it was not detected with a sense probe for *map2k5* (*C*, *D*). Images are lateral views of whole embryos, anterior to the left, dorsal to the top (*A*, *C*), or transverse sections through the spinal cord (sc) trunk regions, dorsal to the top (*B*, *D*). nc, notochord. To confirm *map2k5* expression in the neuronal population, we used FACS to purify EGFP+ neurons at 2 days post fertilization from dissociated cells of *Tg(huC:egfp)* zebrafish, which express EGFP under the control of the neuron-specific *HuC* promoter. Total RNA was isolated from FACS-sorted EGFP+ neurons, and semi-quantitative RT-PCR was performed with PCR primers specific to b-actin, *map2k5*, *huC*, *gfp*, and *flk* (*E*). *beta-Actin*, *map2k5*, neuron-specific *huC*, and *gfp*, but not vessel specific *flk*, were successfully amplified from the sorted neurons (N), whereas *beta-actin*, *map2k5*, *huC*, and *flk*, but not *gfp*, were amplified from whole embryos (AB), indicating that the FACS procedure specifically isolated neurons and that *map2k5* was expressed in whole embryos, including neurons.
