# Supplementary material for: Yeast genetic interaction screen of human genes associated with amyotrophic lateral sclerosis: identification of MAP2K5 kinase as a potential drug target
Source: Genome Res. 2017 Sep;27(9):1487–500. doi: 10.1101/gr.211649.116 (PMC5580709; doi:10.1101/gr.211649.116)
Supplement: Supplemental Material [file supp_gr.211649.116_Supplemental_Fig_S12.pdf]

Supplemental Figure 12

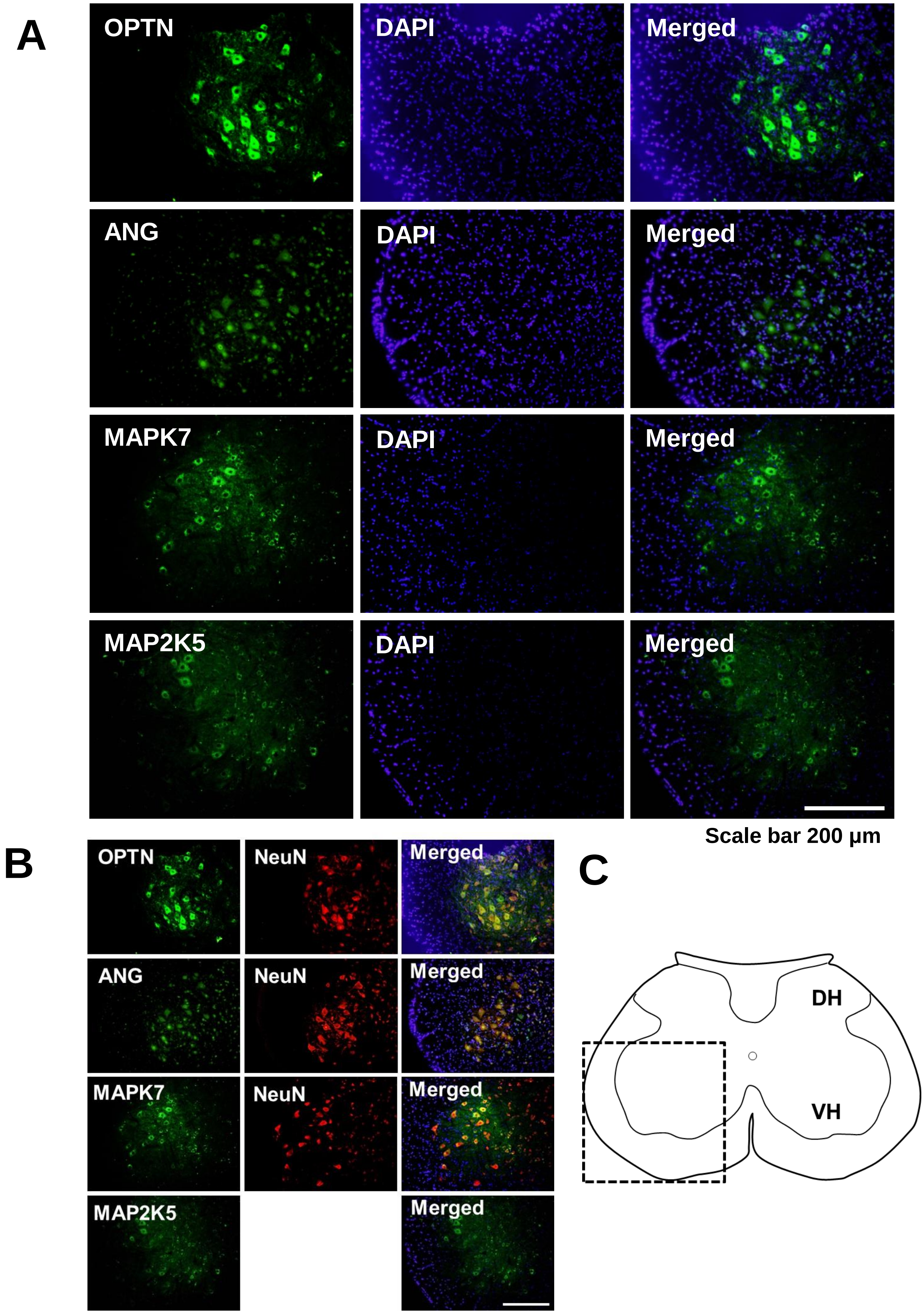

Supplemental Figure 12. Expression of OPTN, ANG, MAPK7, and MAP2K5 in the mouse spinal cord. Immunofluorescence analysis was performed to detect the expression of OPTN, ANG, MAPK7, and MAP2K5 (green) in mouse spinal cord. Nuclei were stained with DAPI (blue), which was merged with images showing OPTN, ANG, MAPK7, and MAP2K5 immunoreactivities (**A**). OPTN, ANG, MAPK7, and MAP2K5 expression was found to be co-localized with NeuN, a neuronal marker (red) (**B**), indicating the neuronal expression of these proteins. Scale bar, 200 µm. Immunofluorescence images were captured from the ventral horn and column of the spinal cord (dotted box) (**C**). DH, dorsal horn; VH, ventral horn.
