# Supplementary material for: Yeast genetic interaction screen of human genes associated with amyotrophic lateral sclerosis: identification of MAP2K5 kinase as a potential drug target
Source: Genome Res. 2017 Sep;27(9):1487–500. doi: 10.1101/gr.211649.116 (PMC5580709; doi:10.1101/gr.211649.116)
Supplement: Supplemental Material [file supp_gr.211649.116_Supplemental_Fig_S13.pdf]

Supplemental Figure 13

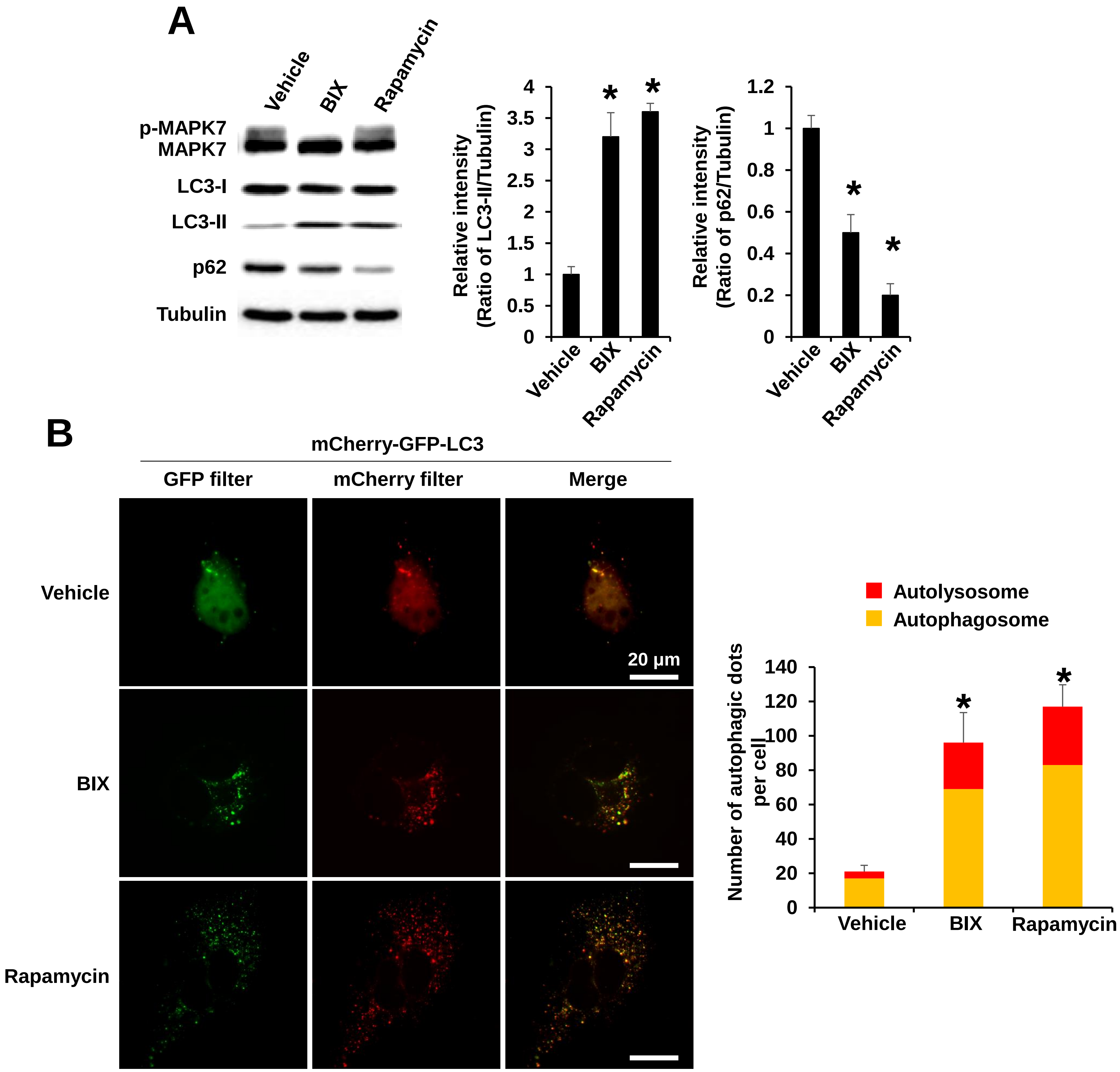

Supplemental Figure 13. Inhibition of MAP2K5 enhances autophagy. (*A*) NIH3T3 cells were treated for 4 hr with 10 μM of BIX 02189 or 200 nM of rapamycin, an autophagy inducer. Protein lysates were subjected to western blot analysis with MAPK7, LC3, and p62 antibodies. Tubulin was detected as a loading control. The results of densitometric analysis (*right*) are presented as the mean ± SD (n = 3); *$p < 0.05$ versus vehicle treatment. (*B*) NIH3T3 cells plated on coverslips were transfected with mCherry-GFP-LC3. After 48 hr of transfection, cells were treated with 10 μM of BIX 02189 or 200 nM of rapamycin for 4 hr. Images show representative fields with autophagosomes (yellow) and autolysosomes (red) (*left*). Scale bar, 20 μm. The graph shows the number of autophagosomes (yellow bar) and autolysosomes (red bar) per cell in the merged images (*right*). Thirty cells were examined from three independent experiments. *$p < 0.05$ versus vehicle.
