# Supplementary material for: Yeast genetic interaction screen of human genes associated with amyotrophic lateral sclerosis: identification of MAP2K5 kinase as a potential drug target
Source: Genome Res. 2017 Sep;27(9):1487–500. doi: 10.1101/gr.211649.116 (PMC5580709; doi:10.1101/gr.211649.116)
Supplement: Supplemental Material [file supp_gr.211649.116_Supplemental_Fig_S14.pdf]

Supplemental Figure 14

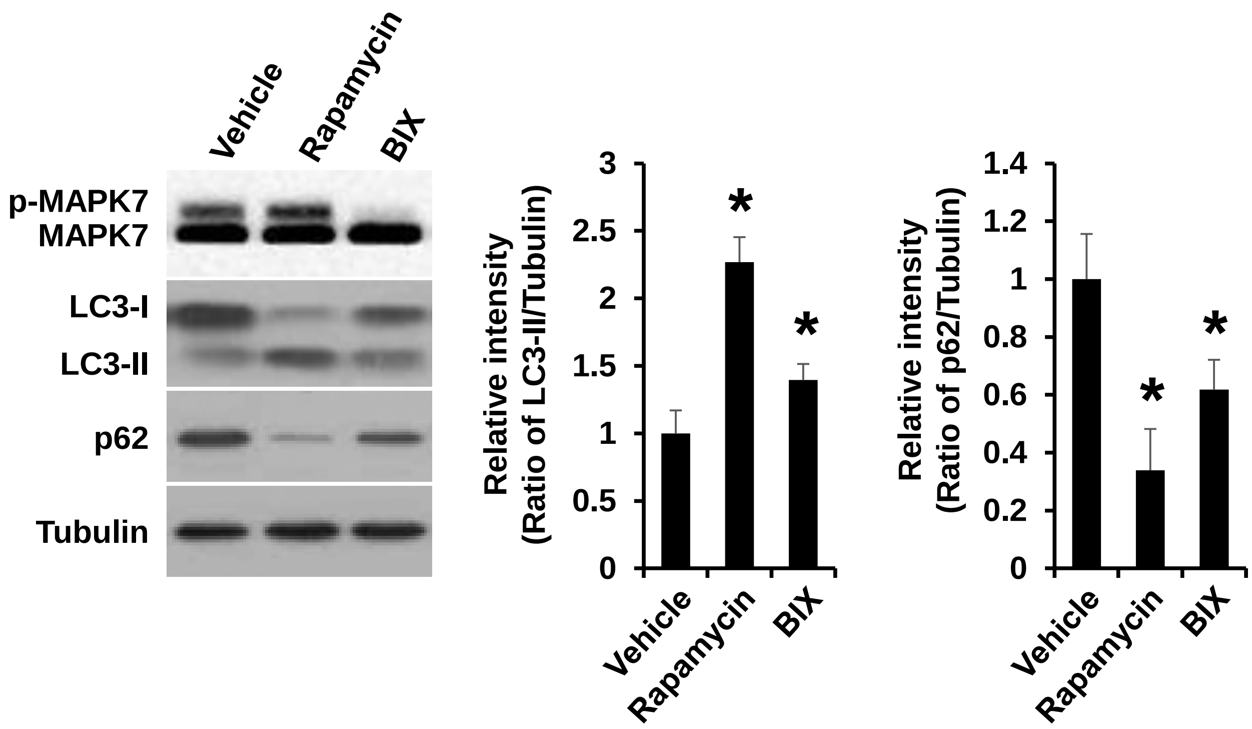

Supplemental Figure 14. Inhibition of MAP2K5 enhances autophagy in motoneuron-like cells. Differentiated NSC-34 mouse motoneuron-like cells were treated for 4 hr with 10 μM of BIX 02189 or 200 nM of rapamycin, an autophagy inducer. Protein lysates were subjected to western blot analysis with MAPK7, LC3, and p62 antibodies. Tubulin was detected as a loading control. The results of densitometric analysis (*right*) are presented as the mean ± SD (n = 3); **p* < 0.05 versus vehicle treatment.
