# Supplementary material for: Yeast genetic interaction screen of human genes associated with amyotrophic lateral sclerosis: identification of MAP2K5 kinase as a potential drug target
Source: Genome Res. 2017 Sep;27(9):1487–500. doi: 10.1101/gr.211649.116 (PMC5580709; doi:10.1101/gr.211649.116)
Supplement: Supplemental Material [file supp_gr.211649.116_Supplemental_Fig_S15.pdf]

Supplemental Figure 15

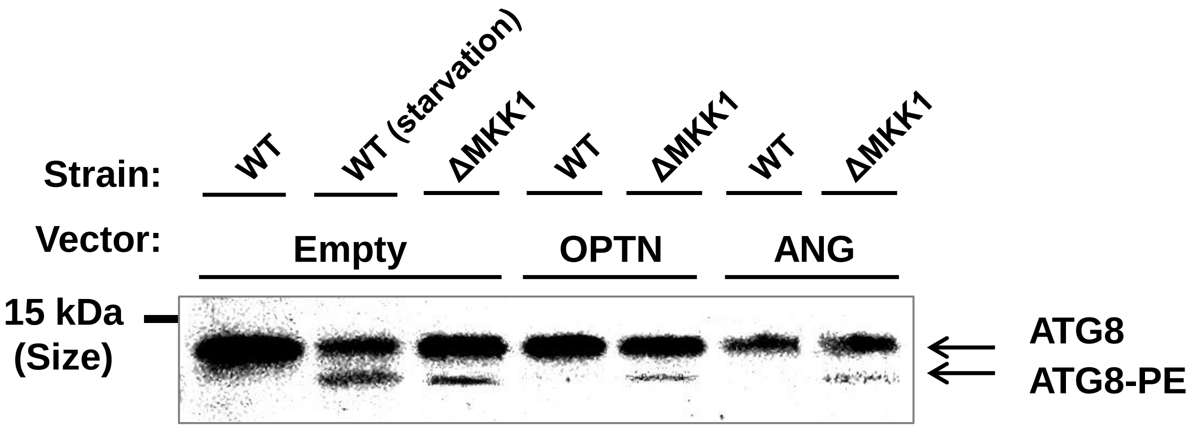

Supplemental Figure 15. MAP2K5 deletion enhances autophagy in yeast. Wild-type yeast strain (BY4742) or MAP2K5 deletion strain (ΔMKK1) was transformed with OPTN or ANG constructs, and then grown to a OD_{600} of 1 in SGal-Leu medium and harvested. For starvation, yeast cells were incubated in SD-N medium for 6 hr. ATG8 lipidation were detected by western blot analysis using an antibody against ATG8. ATG8-PE (phosphatidylethanolamine) was separated from ATG8 by 13.5% SDS PAGE gels containing 6 M urea.
