# Supplementary material for: Yeast genetic interaction screen of human genes associated with amyotrophic lateral sclerosis: identification of MAP2K5 kinase as a potential drug target
Source: Genome Res. 2017 Sep;27(9):1487–500. doi: 10.1101/gr.211649.116 (PMC5580709; doi:10.1101/gr.211649.116)
Supplement: Supplemental Material [file supp_gr.211649.116_Supplemental_Fig_S16.pdf]

Supplemental Figure 16

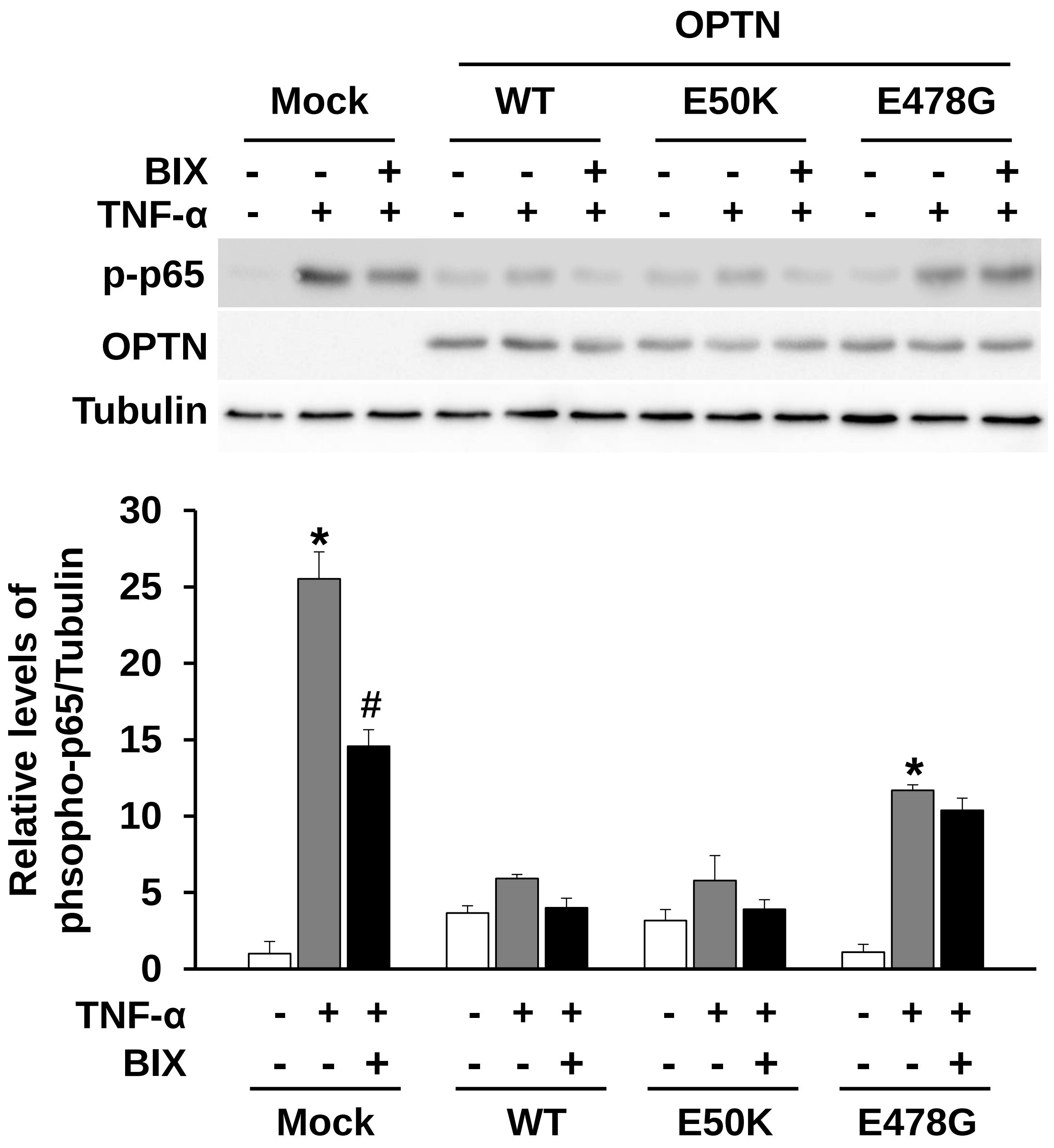

Supplemental Figure 16. Effects of OPTN overexpression and MAP2K5 inhibition on TNF-α induced NF-κB activation. OPTN (wild-type or mutants)-transfected NIH3T3 cells were treated with TNF-α (10 ng/ml) with or without BIX 02189 (10 μM) for 2 hr. The cell lysates were harvested, and phosphorylated p65 subunit of NF-κB was detected by western blot analysis. Tubulin was detected as a loading control. The results are representative of three experiments. The results of densitometric analysis (*lower*) are presented as the mean ± SD ($n = 3$); *$p < 0.05$ versus control. $^{\#}p < 0.05$ versus TNF-α alone.
